# Identification of Trombospondin-1 as a Novel Amelogenin Interactor by Functional Proteomics

**DOI:** 10.3389/fchem.2017.00074

**Published:** 2017-10-09

**Authors:** Angela Capolupo, Chiara Cassiano, Agostino Casapullo, Giuseppina Andreotti, Maria V. Cubellis, Andrea Riccio, Raffaele Riccio, Maria C. Monti

**Affiliations:** ^1^Department of Pharmacy, University of Salerno, Salerno, Italy; ^2^PhD Program in Drug Discovery and Development, University of Salerno, Salerno, Italy; ^3^Istituto di Chimica Biomolecolare, Consiglio Nazionale Delle Ricerche (CNR), Napoli, Italy; ^4^Department of Biology, University of Naples Federico II, Napoli, Italy; ^5^Department of Environmental, Biological and Pharmaceutical Sciences and Technologies, University of Campania Luigi Vanvitelli, Caserta, Italy

**Keywords:** functional proteomics, protein-protein interaction, amelogenin, thrombospondin-1, extracellular matrix proteins, wound healing

## Abstract

Amelogenins are a set of low molecular-weight enamel proteins belonging to a group of extracellular matrix (ECM) proteins with a key role in tooth enamel development and in other regeneration processes, such as wound healing and angiogenesis. Since only few data are actually available to unravel amelogenin mechanism of action in chronic skin healing restoration, we moved to the full characterization of the human amelogenin isoform 2 interactome in the secretome and lysate of Human Umbilical Vein Endothelial cells (HUVEC), using a functional proteomic approach. Trombospondin-1 has been identified as a novel and interesting partner of human amelogenin isoform 2 and their direct binding has been validated thought biophysical orthogonal approaches.

## Introduction

Amelogenins are a set of low molecular-weight enamel matrix proteins belonging to a group of extracellular matrix (ECM) proteins whose genes are greatly preserved, with a key role in tooth enamel advance (Deutsch, 1989; Moradian-Oldak, 2001; Grandin et al., 2012). Amelogenin gene is conserved across vertebrate species, mainly in the N- and C-terminal regions. There are two amelogenin genes (AMELX and AMELY) located on the X and Y chromosomes, respectively. The coding sequence of AMELX had 95.1% identity to that of AMELY (Delgado et al., 2005). The heterogeneity of amelogenins in terms of molecular size and amino acid composition is an outcome of the extended population of their related mRNAs, produced by alternative splicing and translated into a broad mixture of nascent proteins and peptides, among which the most abundant species are the isoforms I and II differing only at the 19–34 peptide level (Svensson Bonde and Bulow, 2012). Amelogenins show a hydrophobic amino terminal fragment and a hydrophilic carboxyl-terminal one, conferring them bipolar properties; moreover, the prevalence of proline, glutamine, leucine, and histidine residues in their aminoacidic composition drives amelogenins to a self-assembling process into large hydrophobic aggregates, which seems to be one of the mechanism engaged in periodontal or tissue regeneration (Khan et al., 2012; Yoshimi et al., 2016). The solubility varies depending on temperature and pH and it is low at neutral pH. Under these conditions, amelogenin species aggregate into larger arrangements, up to form a sort of platelets resembling a stable extra cellular matrix and this transitory complex supplies adhesion sites for cells to connect themselves (Bonde and Bülow, 2012).

In literature two main functions of amelogenins are well-documented: their role in dental enamel formation and their involvement in regeneration processes (Grandin et al., 2012). Amelogenins are mainly involved in dental enamel formation and tooth repair playing an important role in enamel crystal edification, direction, and maturation during crown formation and, thus, they serve as a regulator of enamel biomineralization, endorsing renewal of the periodontium (Grandin et al., 2012). On the contrary, mutations of the human AMELX gene leads to X-linked amelogenesis imperfecta (AI), an inborn disease affecting the tooth development in primary and permanent dentition (Sasaki and Shimokawa, 1995). On this basis, amelogenins are currently considered a relevant adjuvants in the treatment of periodontal defects and they are the main components of the enamel matrix derivative Emdogain® (Emdogain, Straumann), a gel preparation protecting tooth and promoting the regeneration of hard and soft tissues in periodontitis (Sculean et al., 2007).

Besides, based on the pharmacological ability of amelogenins in the successful healing of oral wounds, several studies focused the attention on their potential role in other regeneration processes, such as skin wound healing and angiogenesis (Hoang et al., 2000). Indeed, several studies have shown the helpful role of amelogenins on wound healing and tissue regeneration, activating several crucial cell reactions for tissue restoration and healing, such as proliferation, migration, adhesion and differentiation (Vowden et al., 2006). A care product Xelma® (Xelma, Mölnlycke Health Care) containing amelogenins has been marketed and used in treatment of chronic wounds (Romanelli et al., 2008a,b). Furthermore, periodontal ligament cells (PDL) have been treated with amelogenins for monitoring their effects on cell recruitment, proliferation, and differentiation, indicating an amelogenin-mediated proliferation. This role together with enhanced migration of cells points toward a beneficial wound-healing effects observed for PDL cells stimulated by amelogenins *in vitro* (Davenport et al., 2003). Moreover, a higher proliferation of gingival and dermal fibroblasts following treatment with amelogenins has also been observed (Weinberg et al., 2010). Finally, amelogenins seemed to encourage the angiogenic activity of HUVEC and HMVEC cells stimulating their proliferation and migration also by upregulating mRNA expression of the angiogenic factor *ANG-2* and adhesion molecules *ICAM-1* (Bertl et al., 2009).

In order to increase the knowledge on these regeneration processes, Fukuda and co-workers developed and published a functional proteomic analysis of the amelogenins biological partners. They identified several cytoskeletal proteins and chaperones of heat shock protein 70 (HSP70) family in SaOS-2 osteoblastic cell lysates, as amelogenins partners. Moreover, the proteomic outlines of amelogenin-partners in the membrane fraction of the cell extracts revealed the endoplasmic reticulum (ER)-associated proteins as its best targets, such as glucose-regulated protein 78 (Fukuda et al., 2013).

Since no data are available to unravel amelogenin mechanism of action in chronic skin healing restoration at molecular level, we moved to the full characterization of the interactome of AMELX isoform II, here called AMEL-2, in the secretome and lysate of human umbilical vein endothelial cells (*HUVEC*), chosen as an appropriate *in vitro* model, using a functional proteomic strategy. Functional proteomics is a multi-faced strategy useful to characterize proteins interactions and it has gaining increasing interest to define the “targetome” of macromolecules to elucidate their activity through the affinity with their partners, forming transient and/or stable complexes which in turn mediate biological responses (Raida, 2011). Our best approach is based on the “in solution pull-down” strategy, in which the macromolecule is used as a bait to fish out its specific partners among a complex mixtures, then identified by tandem MS and bioinformatics (Margarucci et al., 2015). Here, we have identified several AMEL-2 potential targets and, among them, we pointed out thrombospondin-1 (TSP-1) as its novel and interesting partner validating the direct binding between the counterparts thought orthogonal biophysical approaches.

## Materials and methods

### Cell culture and secretome

HUVEC cells were grown in plates using EGM2 with 1% (v/v) growth factors (hFGF, VEGF, R3-IGF-1), hydrocortisone, ascorbic acid, glutamine and heparin, keeping them incubated at 37°C in a 5% CO_2_ atmosphere and collected in the first phase of the cell cycle (G1). The cells were harvested by centrifugation (600 g, 5 min), washed twice with PBS (NaH_2_PO_4_ 50 mM and NaCl 150 mM, pH 7.4) and resuspended in 450 μl of PBS at 0.1% of Igepal together with 50 μl of protease inhibitor cocktail (AESBF (4- (2-Aminophenylbenzenesulfonyl fluoride hydrochloride), aprotinin, bovine hydrogen chloride, E-64 [N- (trans-epoxy succinyl] -L-leucine, 4-guanidiobutylamide, EDTA, salt of leupeptine bisulfate) to avoid protein degradation. The resuspended pellet was subjected to homogenization by a manual “Dounce” homogenizator and centrifuged at 10,000 rpm for 5 min at 4°C (Centrifuge 5424-R, Eppendorf). Protein-rich supernatant was extracted and thus separated from insoluble pellets, consisting of membranes and cytoplasmic organelles. The concentration of protein lysate was estimated by Bradford assay and diluted to obtain a concentration of 1 mg/ml (Bradford, 1976).

The secretome was harvested from the cultured cells and concentrated using an Amicon membrane (Millipore); protein concentration was evaluated by Bradford's assay to obtain a final value of 6 mg/ml.

### Cloning, expression, and purification of recombinant AMEL-2

AMEL-2 was a kind gift of Prof Leif Bülow (Department of Pure and Applied Biochemistry, Lund University, Lund, Sweden) and it was expressed and purified according to the protocol outlined by Svensson Bonde and Bülow (2012).

### MALDI-TOF analysis

AMEL-2 sample at a concentration of 12.5 nM (1 μl) was mixed on the spot of the MALDI plate with 1 μl of the saturated CHCA matrix solution (Sigma Aldrich) in 50% ACN, 50% H_2_O, 0.1% TFA and subjected to mass-spectrometric analysis using MALDI-TOF (MALDI micro MX, Waters) to verify its molecular weight. MALDI analysis was conducted in linear mode, with laser values ranging from 250 to 280 arbitrary units. As calibrant, a mixture of lysozyme and albumin from bovine serum was used.

### Peptide mapping of AMEL-2

One microgram of AMEL-2 were incubated in AMBIC 50 mM with 10 ng of Tryp / Lys-C (1:100) (Promega) in AMBIC (NH_4_HCO_3_) 50 mM at 37° C for 4 h. The peptide mixture obtained by proteolytic digestion was measured by MALDI-TOF (MALDI micro MX, Waters) using saturated CHCA matrix solution (Sigma Aldrich) in 50% ACN, 50% H_2_O, 0.1% TFA. 1 μl of the peptide mixture was mixed with 1 μl of matrix on the spot of the sample holder plate. Mass spectra were acquired in positive linear or reflectron mode.

### Biotinylation of AMEL-2 functional groups

Five micromolar of AMEL-2 and 75 μM (1:15) sulfo-NHS-SS-biotin (Thermo Scientific) were incubated in NaHCO_3_ 50 mM and monitored by MALDI-TOF mass spectrometry as reported before at different time intervals *t* = 0 min; *t* = 5 min; *t* = 15 min. The biotinylation step was followed by quenching with 0.05% acetic acid. The concentration and purification steps of the samples were carried out on centricon (Millipore) with a 5,000 Da cut-off centrifuge (Centrifuge 5424-R, Eppendorf) at 10,000 rpm at 4°C for 15 min. SDS-PAGE at 15% of acrylammide was then run and colored by Comassie Brillant Blue staining.

### Combination of affinity chromatography and proteomic analysis

AMEL-2-biotin adduct (1 or 3 nmol) and the control linker were distinctly incubated with 1 mg of HUVEC cells lysate or secretome, under continuous shaking (1 h, 4°C) and then, 20 μL of streptavidin modified beads (Pierce) were added and left for 1 h at 4°C. The beads were centrifuged (865 g, 1 min,4°C) and rinsed six times with PBS (pH 7.4). The proteins interacting with AMEL were eluted by adding 35 μL of Laemmly buffer for 30 min at 30°C, separated on 12% SDS-PAGE and stained with Coomassie G-250 (Bio-Rad, Hercules, CA). SDS-PAGE gel lanes were divided in several pieces and digested. The procedure has been done twice with opportune controls as the matrix bearing the linker without any protein and the sole EGM2. Each gel piece was rinsed with water and CH_3_CN and exposed to *in situ* breakdown as described by Shevchenko et al. (2006). Concisely, each gel piece was treated with 10 mM 1,4-dithiothreitol (DTT) and then with 54 mM iodoacetamide, rinsed and rehydrated in trypsin solution (12 ng/mL) on ice for 1 h. Following AMBIC treatment (30 μL, 50 mM, pH 7.5), protein breakdown was carried out overnight at 37°C. The solution was gathered and peptides were extracted from the gel pieces using 100% CH_3_CN. The peptide sample was dehydrated and suspended in formic acid (FA, 10%) before MS analysis. The peptide mixture (5 μL) was introduced in a nano-ACQUITY UPLC system (Waters). Peptides were separated on a 1.7 mm BEH C18 column (Waters) at a flow rate of 400 nL/min. Peptide elution was achieved with a linear gradient (solution A: 95% H_2_O, 5% CH_3_CN, 0.1% FA; solution B: 95% CH_3_CN, 5% H_2_O, 0.1% FA); 15–50% B over 55 min). MS and MS/MS data were acquired on a LTQ XL mass spectrometry system (ThermoScientific). The five most intense doubly and triply charged ions were broken. The MS results were treated by MS Converter software to obtain the peak lists for protein identification. Database searches were performed on the Mascot server (http://www.matrixscience.com/). The SwissProt database was employed [settings: trypsin with possible two missed cleavages; carbamidomethyl (C) as fixed modification and oxidation (M) and phosphorylation (ST); peptide tolerance 80 ppm; MS/MS tolerance 0.8 Da].

### STRING analysis

STRING analysis (Search Tool for the Retrieval of Interacting Genes/Proteins, http://string.embl.de/) have been carried out using STRING-10 server to predict the protein-protein interaction (Sharma et al., 2016a,b; Sharma and Bisht, 2017a,b) of AMEL targets. STRING database employs a mixture of prediction approaches and an combination of experimental data (neighborhood, gene fusion, co-expression, experiments, databases, text mining, co-occurrence). Network was completed at 0.4 confidence level.

### Validation of the interaction between AMEL-2 and TSP-1 by western blotting and surface plasmon resonance

Proteins eluted from the above-described experiments were separated on 12% SDS-PAGE and moved to a nitrocellulose membrane. The membrane was put for 1 h in a blocking solution made by 25 mM Tris pH 8, 125 mM NaCl, 0.05% Tween-0, 5% non-fat dried milk, primary antibodies raised against TSP-1 (1:500; Novus Biologicals). The recognition of specific epitopes was favorite overnight, at 4°C. Then, membrane was put for 1 h with an anti-rabbit peroxidase-conjugated secondary antibody (1:5,000) (Sigma-Aldrich). TSP-1 was detected by a chemo-luminescence detection system. AMEL-2 was immobilized onto a Biacore 3,000 (GE Healthcare) using a CM5 sensor chip using standard amine coupling procedures. Phosphate–buffered saline (10 mM Na_2_HPO_4_ and 150 mM NaCl, pH 7.4) was the running buffer. The carboxymethyl dextran surface was activated as reported by the GE Healthcare protocol and AMEL-2 was diluted to a final concentration from 50 nM and injected at flow rate of 5 μl/min. After several injections, a RU of 10,000 was measured. The activated carboxymethyl dextran surface was finally blocked with a 7-min injection of 1.0 M ethanolamine-HCl, pH 8.5, at 5 μl/min. TSP-1 solutions(Abnova; from 50 nM to 1 μM) or alternative BSA and lysozime as negative controls, were prepared in running buffer and injected. Since the binding curve went back to baseline in a reasonable time, no regeneration process was mandatory. The experiments were done at a flow rate of 10 μl/min, using a 3 min injection time. The dissociation time was set at 600 s. Rate constants for associations (ka) dissociations (kd) and the dissociation constants (K_D_) were calculated by using results from injections of all concentrations, using the BIAevaluation software and the 1:1 Langmuir binding model.

## Results

Our process to disclose AMEL-2 action targets has been planned in the next steps: (a) Production of AMEL-2 biotin-tagged specie, (b) Incubation and recovery of its specific partners and identification of the interactome by mass spectrometry and bioinformatics, c) Corroboration of AMEL-2 interactions by bio-orthogonal *in vitro* assays.

### Protein expression, purification, and characterization

**Figure 1:**
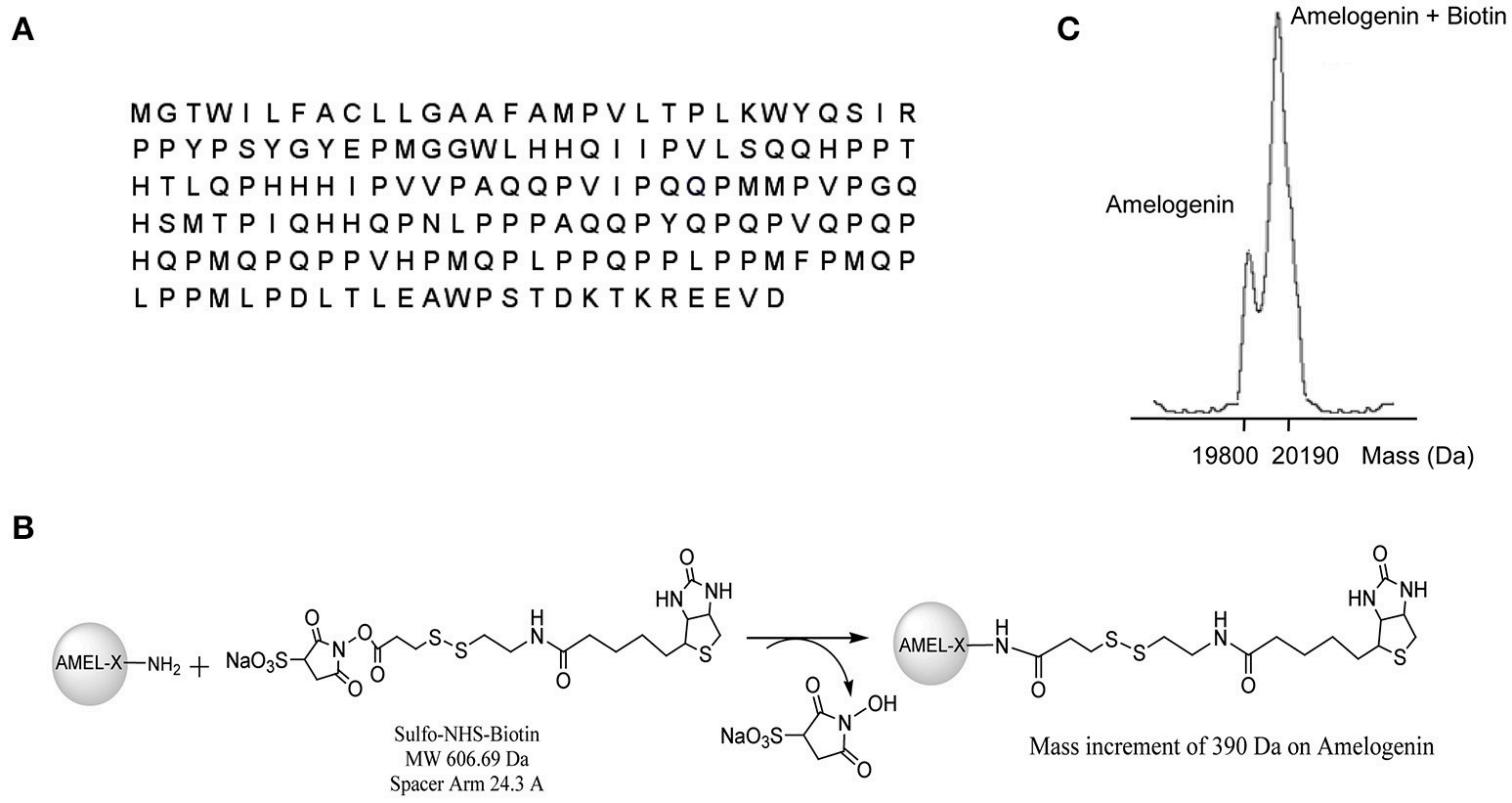
**(A)** reports AMEL-2 sequence. **(B)** Shows the reaction pathways of AMEL-2 with NHS activated S–S–biotin to obtain the NHS–biotin adduct used in functional proteomics experiments. **(C)** Shows MALDI MS analysis of intact AMEL-2 after the reaction with NHS activated S–S–biotin.

A fraction of the expressed AMEL-2 (Figure 1A for sequence, UNIPROT code Q99217, see isoform 2) was measured by MALDI-MS giving a MW of around 19,800 Da, compatible with the isoform II. Protein analysis was performed by digestion with trypsin and MALDI analysis, and three protein fragments were measured and recognized as the 1-24 and 25-168 and 25-170 residues (Supplementary Figure S1), covering almost the entire sequence of AMEL-2.

### Generation of mono-biotinylated AMEL-2

As a first step, AMEL-2 was treated with a N-hydroxysuccinimide activated S–S biotin linker, providing the AMEL-2–biotin derivative reported in Figure 1B. The reaction was monitored by MALDI-MS and tuned with different molar ratios of the reagents and incubation time, with the best results with a molar excess of biotin of 20 times over AMEL-2 (AMEL-2:biotin 1:20) and 15 min of incubation at room temperature. A mono-modified AMEL-2 species was recovered together with the unreacted species (Figure 1C), following separated from the biotin excess by filtration on a 5 kDa membrane tube, without sample loss (Supplementary Figure S2).

### Affinity chromatography and mass spectrometry

**Figure 2:**
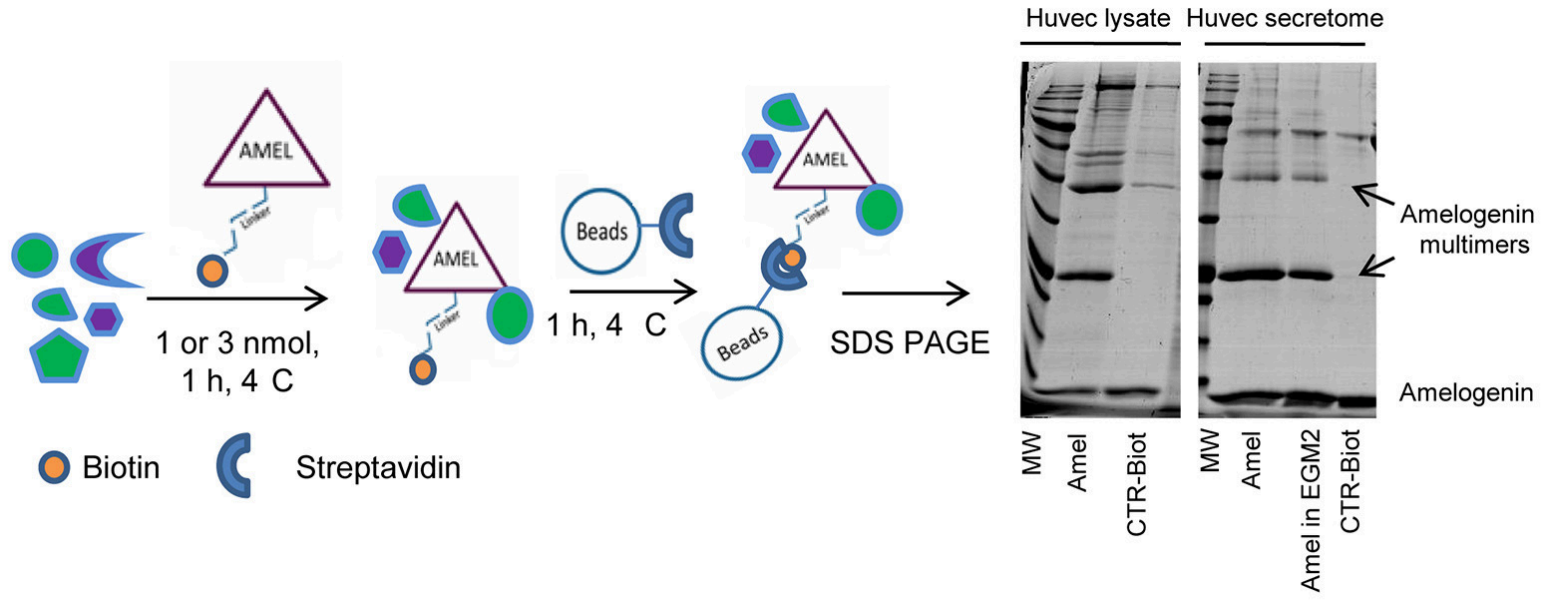
Schematic representation of functional proteomics. AMEL-2-biotin adduct has been incubated with HUVEC lysates or secretome and the protein together with its partners was fished out using streptavidin modified beads. All proteins were eluted and loaded on a 12% SDS-PAGE together with opportune controls. Since AMEL-2 aggregates at neutral pH, the visible multimers were identified as amelogenin aggregates.

Samples of HUVEC protein extracts and the corresponding secretomes were separately incubated with the biotinylated AMEL-2 (1 and 3 nmol, respectively) to endorse the interaction among the protein and its potential target(s) in solution. As control experiments, a sample of the biotin linker modified with ethanolamine was incubated in the same conditions, and a sample of EGM2 culture medium was incubated with the biotinylated AMEL-2. After 1 h, all the parallel experiments were treated with streptavidin-bearing matrix beads to recover, taking advantage of the strong biotin/streptavidin affinity, the biotinylated adducts together with their interactome. Then, the resins were extensively washed to remove the aspecific bound proteins, whereas the tightly bound interactors were released using a Laemmly buffer solution. The eluted protein mixtures were resolved by 12% SDS-PAGE (Figure 2), and gel lanes were divided in pieces and exposed to *in situ* digestion using trypsin (Shevchenko et al., 2006) The tryptic mixtures were investigated through nano-flow RP-HPLC/MSMS and protein identification was carried out presenting the MSMS peak lists to the Mascot database analysis. A refined list of the AMEL-2 partners was obtained after filtering all the hits in common with the control experiments, removing all the proteins with an incongruous molecular weight, all the proteins with a Mascot score less than 40 and all the proteins identified with less than 10 matched peptide signals (Table 1 and Supplementary Table S1 for details).

**Table 1:** List of AMEL-2 partners found in both HUVEC cell lysate (upper Table) and HUVEC cell secretome (lower Table).

**Accession**	**Score**	**Mass**	**Matches**	**Pep(sig)**	**Sequences**	**Seq(sig)**	**emPAI**	**Description**
TBB5_HUMAN	706	50,095	48	29	24	18	6.22	Tubulin beta chain
TBB4B_HUMAN	659	50,255	41	27	23	19	6.16	Tubulin beta-4B chain
FLNB_HUMAN	357	280,157	49	18	48	18	0.36	Filamin-B
TBB6_HUMAN	279	50,281	21	16	15	13	2.72	Tubulin beta-6 chain
MYH9_HUMAN	243	227,646	39	14	36	13	0.31	Myosin-9
FAS_HUMAN	188	275,877	31	13	31	13	0.25	Fatty acid synthase
ENOA_HUMAN	186	47,481	12	7	11	7	1.00	Alpha-enolase
ATPB_HUMAN	145	56,525	14	8	13	8	0.95	ATP synthase subunit beta
EF2_HUMAN	134	96,246	22	8	21	8	0.48	Elongationfactor2
RACK1_HUMAN	89	35,511	11	7	9	6	1.21	Guanine nucleotide-binding protein subunit beta-2-like 1
PDIA1_HUMAN	89	57,480	14	4	14	4	0.39	Protein disulfide-isomerase
PRDX1_HUMAN	87	22,324	13	6	12	6	0.52	Peroxiredoxin-1
TSP1_HUMAN	46	133,291	7	2	7	2	0.07	Thrombospondin-1
FINC_HUMAN	495	266,052	47	19	46	19	0.40	Fibronectin
TSP1_HUMAN	200	133,291	18	10	16	10	0.43	Thrombospondin-1

*For each protein, the following parameters are reported: Mascot score (Score), molecular weight (mass), number of matched peptides (Matches) and unique peptides used in the identification process [Match(sig)], number of sequences (Sequences) and the number of significant distinct sequence matches in the protein identification process [Seq(sig)] and the relative quantization of each protein (emPAI)*.

### String analysis

**Figure 3:**
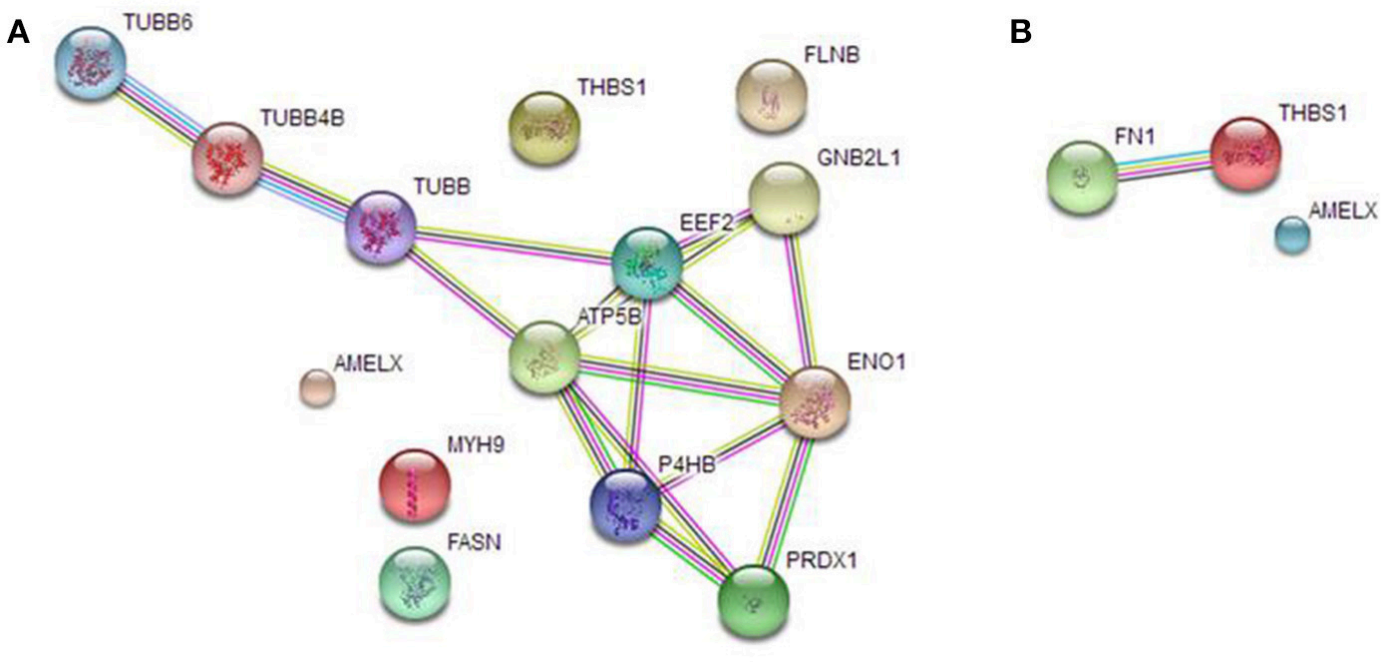
STRING analysis carried out on the putative AMEL-2 partners identified by proteomics both in HUVEC lysates **(A)** and secretome **(B)** revealing protein-protein interaction.

We analyzed AMEL-2 targets, identified by proteomics both in cell lysate (Figure 3A) and secretome (Figure 3B), using STRING-10 software with an intermediate confidence threshold of 0.4 and build an interactome network to find out the known protein-protein interaction (PPIs) and predict functional associations. First of all, actually no interactions of AMEL-2 are reported with its fished out proteins. It has to notice that Fukuda et al. reported the interaction of amelogenin with similar cytoskeletal proteins so it can be feasible to postulate a direct interaction of AMEL-2 with reported tubulins which are involved in an extended interaction pathway, comprising Filamin B, Myosin 9, ATP synthase and others. On the other side, the interaction between TSP-1 and fibronectin, which are the unique two proteins fished out in cell secretome, is very well-documented in STRING. Both are high-molecular weight adhesive glycoproteins, mainly found in the ECM and secreted by various cells, primarily fibroblasts, and they assembled into an insoluble matrix in a complex cell-mediated process.

### Validation of the interaction between AMEL-2 and its counterpart TSP-1

**Figure 4:**
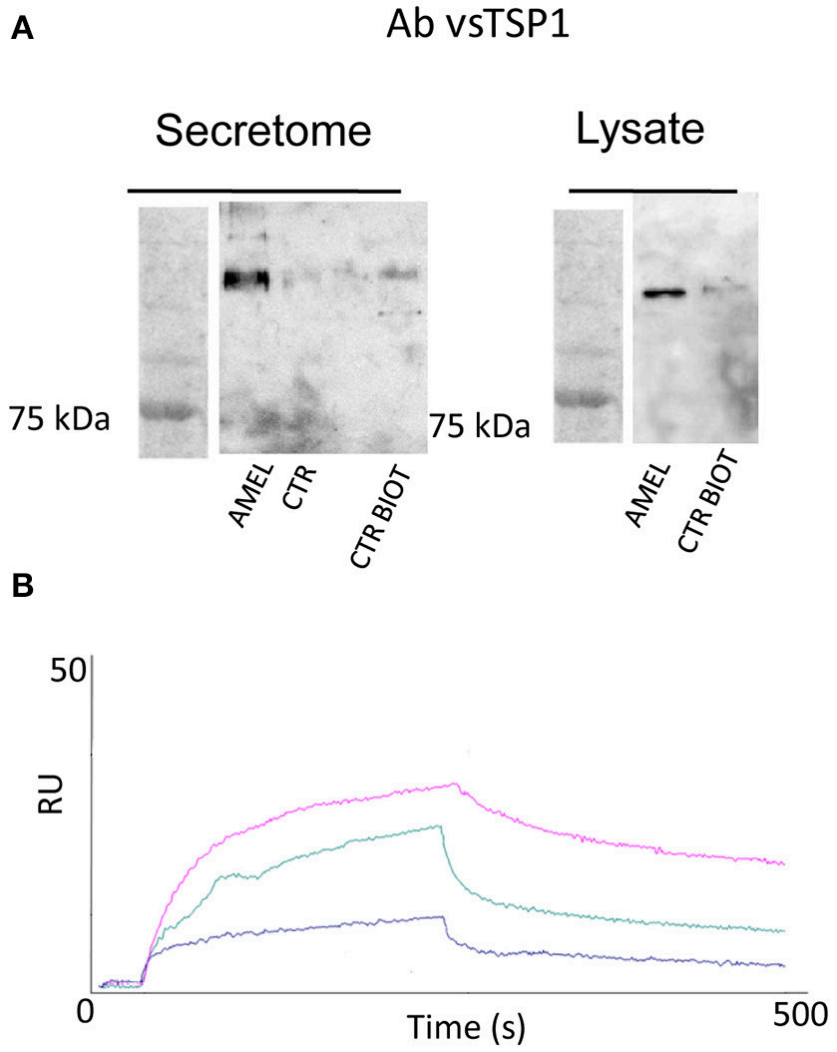
Western blot analysis of TSP-1 protein eluted by AMEL-2 fishing and using opportune control beads incubated with HUVEC cell secretome and lysates **(A)**. SPR sensorgrams obtained on a AMEL-2-modified sensor chip at different concentrations (50 to 5,000 nM) of free TSP-1 **(B)**.

As described in Table 1, a few proteins were identified as the most appropriate partners of AMEL-2 and, among them, the trombospondin-1 protein (TSP-1), fished out by AMEL-2 both in the cell lysate and, mostly, in the secretome was selected for further investigation. The interaction of AMEL-2 with TSP-1 was then confirmed by immune-blotting analysis both the interactome fished out by AMEL-2 on the lysate and the secretome of the HUVEC cells. As clearly reported in Figure 4A, a strong band was evident on the gel lanes corresponding to the experiment with the biotilnylatedAMEL-2, while no signal was detected in the control lanes. Then, surface plasmon resonance was used for corroborating proteomic data and to give evidence of a direct interaction between AMEL-2 and TSP-1, measuring their binding affinity, while bovine serum albumin and lysozime were used as negative controls (Supplementary Figure S3) AMEL-2 was immobilized on a CM-5 sensor chip prior to the injection of TSP-1 at different concentrations (from 50 to 5,000 nM, Figure 4). Sensorgram analysis gave a dissociation constant (K_D_) of the AMEL-2-TSP-1 complex as 878 (±125) nM, while no binding was observed for BSA and lysozime.

## Discussion

Matricellular proteins are extracellular biomolecules acting through their multiple connection to different ligands (Hugo et al., 1995): this is the key feature of these proteins, elucidating their typical variety of functions. These interactions can take concurrently place having various effects on the receptor/ligand equilibrium having different downstream signals, such as the activation of receptors, the arrangement of macro complexes, inhibition of growth factors, change of protein localization and so on (Resovi et al., 2014). In this scenario, a complete mapping of the amelogenin interactome is required to elucidate its functional role. In addition, the identification of new ligands and protein-protein networks might shed light on its mechanism of action and/or point to new roles for amelogenin in physiological and pathological processes. Amelogenin partners were well-characterized in osteoblast activation by coupling affinity chromatography with proteomics in fractionated SaOS-2 osteoblastic cell lysate (Fukuda et al., 2013). In the cytoplasm, the amelogenin interacting proteins were mainly cytoskeletal and heat shock proteins as heat shock protein 70 (HSP70) whereas in the membrane fraction were mainly endoplasmic reticulum (ER)-associated proteins, such as glucose-regulated protein 78 (Grp78/Bip). These data were promising for understanding the cellular and molecular bases of amelogenin mechanisms in periodontal tissue regeneration.

On the contrary, looking at amelogenin task in chronic skin healing reinstatement, no data were available to unravel its mechanism of action. In this study, the HUVEC cells were used as an opportune model of healing restoration and AMEL-2 partners were disclosed both in the cell lysates and in secretomes through a functional proteomic approach. Several proteins were pulled-down and identified in the cell lysates as potential AMEL-2 targets, mainly cytoskeletal proteins, such as tubulins, myosin and filamin, that were expected on the basis of previous studies (Fukuda et al., 2013).

These cytoskeletal proteins are strictly connected giving a network of protein-protein interaction (PPI see Figure 3) as also suggested by STRING interactome analysis and they could be involved into the wound healing process modulating cell locomotion, crucial for repair of injury and for immune response.

Actin polymerization represents a central node for the formation of protrusive structures that drive cell mobility (Ridley et al., 2003). Actin dynamics is an orchestrated cascade of biochemical events that involves a number of regulatory signals and several actin-binding proteins (Lambrechts et al., 2004). Besides, myosin motor proteins play a pivotal role in cells spreading since actin/myosin stress fibers generate the contractile forces that allow the detachment of the rear of the moving cell and, in turn, cell translocation (Lauffenburger and Horwitz, 1996). Specific roles of different myosin isoforms have been reported. In particular, Myosin 9, also called Myosin IIA which targets AMEL-2, is involved in cytoskeleton reorganization, focal contacts formation and lamellipodial retraction negatively affecting cell migration and regulating microtubule dynamics, maintaining a balance between the actomyosin and microtubules. Furthermore, Myosin IIA depletion from human cancer cells resulted in an increased rate of wound closure (Even-Ram et al., 2007). Filamins function as scaffold proteins connecting actin filaments to various cell membrane constituents. Both Filamin A and Filamin B are enriched in focal adhesion which mechanically and biochemically links actin bundles to the ECM acting on cell migration (Tosse et al., 2001). The interaction of AMEL-2 with protein belonging to the tubulin family may also play a role in wound healing. Although the exact mechanism of tubulin action in this process remains debated, the microtubules dynamics regulates many aspects of cell migration (Ganguly et al., 2012; Charafeddine et al., 2016).

AMEL-2 is postulated to interact also with Protein disulfide-isomerase (PDIA-1) in the present work. PDI is a member of the thioredoxin superfamily mainly localized in endoplasmic reticulum, where it ensures proper disulfide bond formation in newly synthesized proteins and endowed with three catalytic activities including, thiol-disulfide oxi-reductase, disulfide isomerase and redox-dependent chaperone (Ali Khan and Mutus, 2008). PDI was also found to localize on the surface of platelets, endothelial cells, and leukocytes and to be released into the extracellular environment by endothelial cells and bound platelets during thrombus formation (Flaumenhaft, 2013) Several lines of evidence have raced the idea that PDI is directly involved in thrombus formation by regulating the activation of proteins required in this process. Interestingly, although this interaction was not fully investigated, TSP-1 was identified as a potential PDI substrate (Bowley et al., 2017). Moreover, the ability of PDI to catalyze the formation of TSP-1-Thrombin–Antithrombin III complex was shown *in vitro* (Milev and Essex, 1999).

A fine tuning of Peroxiredoxin 1 activity, strictly connected to extracellular hydrogen peroxide (H_2_O_2_) levels, was suggested to play a role in wound healing process. H_2_O_2_ is normally produced by mammalian cells as result of metabolic processes. In specific conditions, such as wound healing, the amount of produced H_2_O_2_ increases considerably, representing a protection against pathogens, but its toxic accumulation must be prevented by enzymes also belonging to the peroxiredoxin family (Schäfer and Werner, 2008). Low levels of H_2_O_2_ are essential for propagation of intracellular signaling triggered by growth factor and required for re-epithelization. Peroxiredoxin 1 resulted inactivated at the margin of healing cutaneous wounds in mice to allow wound repair mediated by H_2_O_2_ (Woo et al., 2010).

Alpha-enolase (ENO1) is another AMEL-2 interactor, involved in regeneration processes. Indeed, ENO1 is a key glycolytic enzyme that catalyzes the dehydration of 2-phospho-D-glycerate to phosphor-enol-pyruvate. Besides its cytoplasmic localization, ENO1 is exposed on the cell surface where acts as a plasminogen receptor (Miles et al., 1991). When translocated to the cell surface, ENO1 contributes to pericellular plasmin generation that in turn plays a crucial part in degradation of fibrin provisional matrix and other ECM components. The ENO1 increased cell-surface expression was suggested to have an important role also in inflammatory cell invasion (Wygrecka et al., 2009).

Guanine nucleotide-binding protein subunit beta-2-like 1, commonly defined as RACK1, is a WD40 repeat protein that showed a wide variety of cellular interactors involved in determining the physiology of healing wounds. Many other biological partners may be involved in RACK1 dependent regulation of cell migration (Hermanto et al., 2002; Cox et al., 2003; Songhai et al., 2008).

Among all potential AMEL-2 targets, we pointed out our attention mainly on TSP-1. This protein was fished out by AMEL-2 both in HUVEC cell lysates and secretomes, as the unique common partner. Moreover, in the HUVEC secretomes fibronectin (FINC) was identified as an AMEL-2 interacting protein. Since fibronectin is a well-known specific target of TSP-1 as also reported by interactome analysis by STRING, we were interested to better analyze this PPI pathway. Indeed, both TSP-1 and FINC are extra-cellular matrix glycoproteins with adhesive domains interacting between each other.

On this basis, we first validated our proteomic results by immunoblotting analysis and then we proved the direct interaction between TSP-1 and AMEL-2 by surface plasmon resonance, measuring a dissociation constant of around 800 nM. This data excluded a potential role of fibronectin as connection between AMEL-2 and TSP-1 interaction, instead giving evidence for the first time of TSP-1 as new specific AMEL-2 partner.

TSP-1 is a multifunctional extra-cellular matrix glycoprotein produced by several cell types implicated in wound repair, such as keratinocytes, fibroblasts, endothelial cells, and macrophages (Dipietro et al., 1996). Thrombospondins (TSPs) are a small group of secreted, modular glycoproteins. TSP-1 is the most considered element of this class, together with TSP-2, and it is found inside and outside the cell as a 450-kDa trimer. TSP-1 is a prototype matricellular protein that can physically interact with a variety of ligands (Sage and Bornstein, 1991), and seems to be caught up in the regulation of several processes in the healing of skin wounds (Kyriakides and MacLauchlan, 2009). Its mechanism of action has not be yet fully elucidated and controversial data are present in literature: for instance, the ability of TSP-1 to serve as an adhesive substratum was reported in TSP-1 assembly by macrophages facilitating the repair process and providing evidence that TSP-1 production is an important component of optimal wound healing (Dipietro et al., 1996). However, in other cases, TSP-1 supports cell attachment but not spreading and is counter adhesive when added to an adhesive protein, such as fibronectin (Sage and Bornstein, 1991). Moreover, in endothelial cell, numerous investigations have documented that TSP-1 inhibits endothelial cell chemotaxis, adhesion, attachment, and capillary growth *in vitro* (Murphy-Ullrich and Höök, 1989). Finally, wound healing studies in TSP1-null mice specified a tardy repair, characterized by loosely compacted and disorganized granulation tissue (Agah et al., 2005).

On this basis, the finding of a direct interaction between AMEL-2 and TSP-1 can represent an important result to give a better understanding about the role of AMEL-2 in the complex network of the regeneration process. We can postulate that AMEL-2 interaction with TSP-1 may modulate the recognition between this latter protein and FINC which induces a TSP-1 conformation change related to its stabilization and its incorporation into ECM (Tan and Lawler, 2009). This, in turn, may alter the regeneration process and wound healing. This result open the way to following studies to evaluate the potential mechanism of amelogenins in the wound healing process.

## Author contributions

AR, MC, AGC, and MM conceived and designed the experiments; ANC, CC, and GA performed the experiments; ANC, CC, RR, and MM analyzed the data; RR and MM wrote the paper.

### Conflict of interest statement

The authors declare that the research was conducted in the absence of any commercial or financial relationships that could be construed as a potential conflict of interest.

## References

[B1] Agah A. Kyriakides T. R. Bornstein P. (2005). Proteolysis of cell-surface tissue transglutaminase by matrix metalloproteinase-2 contributes to the adhesive defect and matrix abnormalities in thrombospondin-2-null fibroblasts and mice. Am. J. Pathol. 167, 81–88. 10.1016/S0002-9440(10)62955-0 15972954 PMC1603445

[B2] Ali Khan H. Mutus B. (2008). Protein disulfide isomerase a multifunctional protein with multiple physiological roles. Front. Chem. 2:70. 10.3389/fchem.2014.00070 25207270 PMC4144422

[B3] Bertl K. Bruckmann C. Dard M. Andrukhov O. Matejka M. Rausch-Fan X. (2009). Effects of enamel matrix derivative on proliferation/viability, migration, and expression of angiogenic factor and adhesion molecules in endothelial cells *in vitro*. J. Periodontol. 80, 1622–1630. 10.1902/jop.2009.090157 19792852

[B4] Bonde J. S. Bülow L. (2012). Use of human amelogenin in molecular encapsulation for the design of pH responsive microparticles. BMC Biotechnol. 12:25. 10.1186/1472-6750-12-25 22630169 PMC3403901

[B6] Bowley S. R. Fang C. Merrill-Skoloff G. Furie 1 B. C. Furie B. (2017). Protein disulfide isomerase secretion following vascular injury initiates a regulatory pathway for thrombus formation. Nat. Commun. 8:14151. 10.1038/ncomms14151 28218242 PMC5321760

[B7] Bradford M. M. (1976). A rapid and sensitive method for the quantitation of microgram quantities of protein utilizing the principle of protein-dye binding. Anal. Biochem. 72, 248–256. 10.1016/0003-2697(76)90527-3 942051

[B8] Charafeddine R. A. Nosanchuk J. D. Sharp D. J. (2016). Targeting microtubules for wound repair. Adv. Wound Care 5, 444–454. 10.1089/wound.2015.0658 27785378 PMC5067841

[B9] Cox E. A. Bennin D. Doan A. T. O'Toole T. Huttenlocher A. (2003). RACK1 regulates integrin-mediated adhesion, protrusion, and chemotactic cell migration via its Src-binding site. Mol. Biol. Cell. 14, 658–669. 10.1091/mbc.E02-03-0142 12589061 PMC149999

[B10] Davenport D. R. Mailhot J. M. Wataha J. C. Billman M. A. Sharawy M. M. Shrout M. K. (2003). Effects of enamel matrix protein application on the viability, proliferation, and attachment of human periodontal ligament fibroblasts to diseased root surfaces *in vitro*. J. Clin. Periodontol. 30, 125–131. 10.1034/j.1600-051X.2003.00150.x 12622854

[B11] Delgado S. Girondot M. Sire J. Y. (2005). Molecular evolution of amelogenin in mammals. J. Mol. Evol. 60, 12–30. 10.1007/s00239-003-0070-8 15696365

[B12] Deutsch D. (1989). Structure and function of enamel gene products. Anat. Rec. 224, 189–210. 10.1002/ar.1092240209 2672884

[B13] Dipietro L. A. Nissen N. N. Gamelli R. L. Koch A. E. Pyle J. M. Polverini P. J. (1996). Thrombospondin 1 synthesis and function in wound repair. Am. J. Pathol. 148, 1851–1860. 8669471 PMC1861632

[B14] Even-Ram S. D. Doyle A. Conti M. A. Matsumoto K. Adelstein R. S. Yamada K. M. (2007). Myosin IIA regulates cell motility and actomyosin–microtubule crosstalk. Nat. Cell Biol. 9, 299–309. 10.1038/ncb1540 17310241

[B15] Flaumenhaft R. (2013). Protein disulfide isomerase as an antithrombotic target. *Trends Cardiovasc*. Med. 23, 264–268. 10.1016/j.tcm.2013.03.001 PMC3701031 23541171

[B16] Fukuda T. Sanui T. Toyoda K. Tanaka U. Taketomi T. Uchiumi T.. (2013). Identification of novel amelogenin-binding proteins by proteomics analysis. PLoS ONE 8:e78129. 10.1371/journal.pone.0078129 24167599 PMC3805512

[B17] Ganguly A. Yang H. Sharma R. Patel K. D. Cabral F. (2012). The role of microtubules and their dynamics in cell migration. J. Biol. Chem. 287, 43359–43369. 10.1074/jbc.M112.423905 23135278 PMC3527923

[B18] Grandin H. M. Gemperli A. C. Dard M. (2012). Enamel matrix derivative: a review of cellular effects *in vitro* and a model of molecular arrangement and functioning. Tissue Eng. B Rev. 18, 181–202. 10.1089/ten.teb.2011.0365 22070552

[B19] Hermanto U. Zong C. S. Li W. Wang L. H. (2002). RACK1, an insulin-like growth factor I (IGF-I) receptor-interacting protein, modulates IGF-I-dependent integrin signaling and promotes cell spreading and contact with extracellular matrix. Mol. Cell. Biol. 22, 2345–2365. 10.1128/MCB.22.7.2345-2365.2002 11884618 PMC133698

[B20] Hoang A. M. Oates T. W. Cochran D. L. (2000). *In Vitro* wound healing responses to enamel matrix derivative. J. Periodontol. 71, 1270–1277. 10.1902/jop.2000.71.8.1270 10972642

[B21] Hugo C. Pichler R. Meek R. Gordon K. Kyriakides T. Floege J.. (1995). Thrombospondin 1 is expressed by proliferating mesangial cells and is up-regulated by PDGF and bFGF *in vivo*. Kidney Int. 48, 1846–1856. 10.1038/ki.1995.483 8587244

[B22] Khan F. Li W. Habelitz S. (2012). Biophysical characterization of synthetic amelogenin C-terminal peptides. Eur. J. Oral. Sci. 120, 113–122. 10.1111/j.1600-0722.2012.00941.x 22409217 PMC3306135

[B24] Kyriakides T. R. MacLauchlan S. (2009). The role of thrombospondins in wound healing, ischemia, and the foreign body reaction. J. Cell Commun. Signal. 3, 215–225. 10.1007/s12079-009-0077-z 19844806 PMC2778594

[B25] Lambrechts A. Van Troys M. Ampe C. (2004). The actin cytoskeleton in normal and pathological cell motility. Int. J. Biochem. Cell Biol. 36, 1890–1909. 10.1016/j.biocel.2004.01.024 15203104

[B26] Lauffenburger D. A. Horwitz A. F. (1996). Cell migration: a physically integrated molecular process. Cell 84, 359–369. 10.1016/S0092-8674(00)81280-5 8608589

[B27] Margarucci L. Monti M. C. Tosco A. Esposito R. Zampella A. Sepe V.. (2015). Theonellasterone, a steroidal metabolite isolated from a Theonella sponge, protects peroxiredoxin-1 from oxidative stress reactions. Chem. Commun. (Camb). 51, 1591–1593. 10.1039/C4CC09205H 25503482

[B28] Miles L. A. Dahlberg C. M. Plescia J. Felez J. Kato K. Plow E. F. (1991). Role of cell surface lysines in plasminogen binding to cells: identification of alpha-enolase as a candidate plasminogen receptor. Biochemistry 30, 1682–1691. 10.1021/bi00220a034 1847072

[B29] Milev Y. Essex D. W. (1999). Protein disulfide isomerase catalyzes the formation of disulfide-linked complexes of thrombospondin-1 with thrombin-antithrombin III. Arch. Biochem. Biophys. 361, 120–126. 10.1006/abbi.1998.0963 9882436

[B30] Moradian-Oldak J. (2001). Amelogenins: assembly, processing and control of crystal morphology. Matrix Biol. 20, 293–305. 10.1016/S0945-053X(01)00154-8 11566263

[B31] Murphy-Ullrich J. E. Höök M. (1989). Thrombospondin modulates focal adhesions in endothelial cells. J. Cell Biol. 109, 1309–1319. 10.1083/jcb.109.3.1309 2768342 PMC2115751

[B32] Raida M. (2011). Drug target deconvolution by chemical proteomics. Curr. Opin. Chem. Biol. 15, 570–575. 10.1016/j.cbpa.2011.06.016 21763176

[B33] Resovi A. Pinessi D. Chiorino G. Taraboletti G. (2014). Current understanding of the thrombospondin-1 interactome. Matrix Biol. 37, 83–91. 10.1016/j.matbio.2014.01.012 24476925

[B34] Ridley A. J. Schwartz M. A. Burridge K. Firtel R. A. Ginsberg M. H. Borisy G.. (2003). Cell migration: integrating signals from front to back. Science 302, 1704–1709. 10.1126/science.1092053 14657486

[B35] Romanelli M. Dini V. Vowden P. Ågren M. S. (2008a). Amelogenin, an extracellular matrix protein, in the treatment of venous leg ulcers and other hard-to-heal wounds: experimental and clinical evidence. Clin. Interv. Aging 3, 263–272. 10.2147/CIA.S1846 18686749 PMC2546471

[B36] Romanelli M. Kaha E. Stege H. Wnorowski J. W. Vowden P. Majamaa H.. (2008b). Effect of amelogenin extracellular matrix protein and compression on hard-to-heal venous leg ulcers: follow-up data. J. Wound Care 17, 17–23. 10.12968/jowc.2008.17.1.27916 18210952

[B37] Sage E. H. Bornstein P. (1991). Extracellular proteins that modulate cell-matrix interactions. SPARC, tenascin, and thrombospondin. J. Biol. Chem. 266, 14831–14834. 1714444

[B38] Sasaki S. Shimokawa H. (1995). The amelogenin gene. Int. J. Dev. Biol. 39, 127–133. 7626398

[B39] Schäfer M. Werner S. (2008). Oxidative stress in normal and impaired wound repair. Pharmacol. Res. 58, 165–171. 10.1016/j.phrs.2008.06.004 18617006

[B40] Sculean A. Windisch P. Döri F. Keglevich T. Molnár B. Gera I. (2007). Emdogain in regenerative periodontal therapy. A review of the literature. Fogorv. Sz. 100, 220–232, 211–229. 18078142

[B41] Sharma D. Bisht D. (2017a). Role of bacterioferritin and ferritin in *M. tuberculosis* pathogenesis and drug resistance: a future perspective by interactomic approach. Front. Cell. Infect. Microbiol. 7:240. 10.3389/fcimb.2017.00240 28642844 PMC5462900

[B42] Sharma D. Bisht D. (2017b). Secretory proteome analysis of streptomycin-resistant *Mycobacterium tuberculosis* clinical isolates. SLAS Discov. 1:2472555217698428 10.1177/2472555217698428 28314116

[B43] Sharma D. Lata M. Faheem M. Khan A. U. Joshi B. Venkatesan K.. (2016a). *M. tuberculosis* ferritin (Rv3841): potential involvement in Amikacin (AK) and Kanamycin (KM) resistance. Biochem. Biophys. Res. Commun. 478, 908–912. 10.1016/j.bbrc.2016.08.049 27521892

[B44] Sharma D. Lata M. Singh R. Deo N. Venkatesan K. Bisht D. (2016b). Cytosolic proteome profiling of aminoglycosides resistant *Mycobacterium tuberculosis* clinical isolates using MALDI-TOF/MS. Front. Microbiol. 7:1816. 10.3389/fmicb.2016.01816 27895634 PMC5108770

[B45] Shevchenko A. Tomas H. Havlis J. Olsen J. V. Mann M. (2006). In-gel digestion for mass spectrometric characterization of proteins and proteomes. Nat. Protoc. 1, 2856–2860. 10.1038/nprot.2006.468 17406544

[B46] Songhai C. Fang L. Myung E. S. Fei W. Lixin S. Heidi E. (2008). RACK1 regulates directional cell migration by acting on gβγ at the interface with its effectors PLCβ and PI3Kγ. Mol. Biol. Cell. 19, 3909–3922. 10.1091/mbc.E08-04-0433 18596232 PMC2526680

[B47] Svensson Bonde J. Bulow L. (2012). One-step purification of recombinant human amelogenin and use of amelogenin as a fusion partner. PLoS ONE 7:e33269. 10.1371/journal.pone.0033269 22442680 PMC3307724

[B23] Tan K. Lawler J. (2009). The interaction of Thrombospondins with extracellular matrix proteins. J. Cell Commun. Signal. 3, 177–187. 10.1007/s12079-009-0074-2 19830595 PMC2778591

[B48] Tosse T. P. Condeelis J. Cooley L. Hartwig J. H. Noegel A. Schleicher M. (2001). Filamins as integrators of cell mechanics and signalling. Nat. Rev. Mol. Cell Biol. 2, 138–145. 10.1038/35052082 11252955

[B49] Vowden P. Romanelli M. Peter R. Boström Å. Josefsson A. Stege H. (2006). The effect of amelogenins (Xelma™) on hard-to-heal venous leg ulcers. Wound Repair Regen. 14, 240–246. 10.1111/j.1743-6109.2006.00117.x 16808801

[B50] Weinberg E. Weinberg E. Topaz M. Dard M. Lyngstadaas P. Nemcovsky C.. (2010). Differential effects of prostaglandin E-2 and enamel matrix derivative on the proliferation of human gingival and dermal fibroblasts and gingival keratinocytes. J. Periodont. Res. 45, 731–740. 10.1111/j.1600-0765.2010.01293.x 20682018

[B51] Woo H. A. Yim S. H. Shin D. H. Kang D. Yu D. Y. Rhee S. G. (2010). Inactivation of peroxiredoxin I by phosphorylation allows localized H_2_O_2_ accumulation for cell signalling. Cell 140, 517–528. 10.1016/j.cell.2010.01.009 20178744

[B52] Wygrecka M. Marsh L. M. Morty R. E. Henneke I. Guenther A. Lohmeyer J.. (2009). Enolase-1 promotes plasminogen-mediated recruitment of monocytes to the acutely inflamed lung. Blood 113, 5588–5598. 10.1182/blood-2008-08-170837 19182206

[B53] Yoshimi Y. Kunimatsu R. Hirose N. Awada T. Miyauchi M. Takata T.. (2016). Effects of C-terminal amelogenin peptide on proliferation of human cementoblast lineage cells. J. Periodontol. 87, 820–827. 10.1902/jop.2016.150507 27043257

