# Supplementary material for: Identification of Trombospondin-1 as a Novel Amelogenin Interactor by Functional Proteomics
Source: Front Chem. 2017 Oct 9;5:74. doi: 10.3389/fchem.2017.00074 (PMC5635807; doi:10.3389/fchem.2017.00074)

Supplementary Material

Identification of trombospondin-1 as a novel amelogenin interactor by functional proteomics.

Angela Capolupo, Chiara Cassiano, Agostino Casapullo, Giuseppina Andreotti, Maria Vittoria Cubellis, Andrea Riccio, Raffaele Riccio*^*^* and Maria Chiara Monti^*^

*** Correspondence:** Prof. Raffele Riccio, Prof. Maria Chiara Monti
[riccio@unisa.it](mailto:riccio@unisa.it), [mcmonti@unisa.it](mailto:mcmonti@unisa.it).

**Supplementary Figures**

**Supplementary Figure 1.** Panel A shows the linear MALDI-MS spectrum of the intact human amelogenin isoform-2 in its singly- and double-charged forms. Panel B shows the linear MALDI-MS spectrum of the fragments 25-168 and 25-170 generated from human amelogenin isoform-2 by trypsin cleavage. Panel C shows the reflectron MALDI-MS spectrum of the fragment 1-24 generated from human amelogenin isoform-2 by trypsin cleavage.

**Supplementary Figure 2.** SDS-PAGE separation of AMEL-2 before (AMEL) and after (AMEl-biot) the reaction with NHS-SS-biotin and dialysis process.

**Supplementary Figure 3.** SPR sensorgrams obtained on a AMEL-2-modified sensor chip at different concentrations (50 to 5000 nM) of free bovine serum albumin (on the left) and lysozime (on the right). No binding is confirmed since no association neither dissociation phases were visible.

**Supplementary Table S1**

**Identification of AMEL-2 partners by Mascot Search**

**
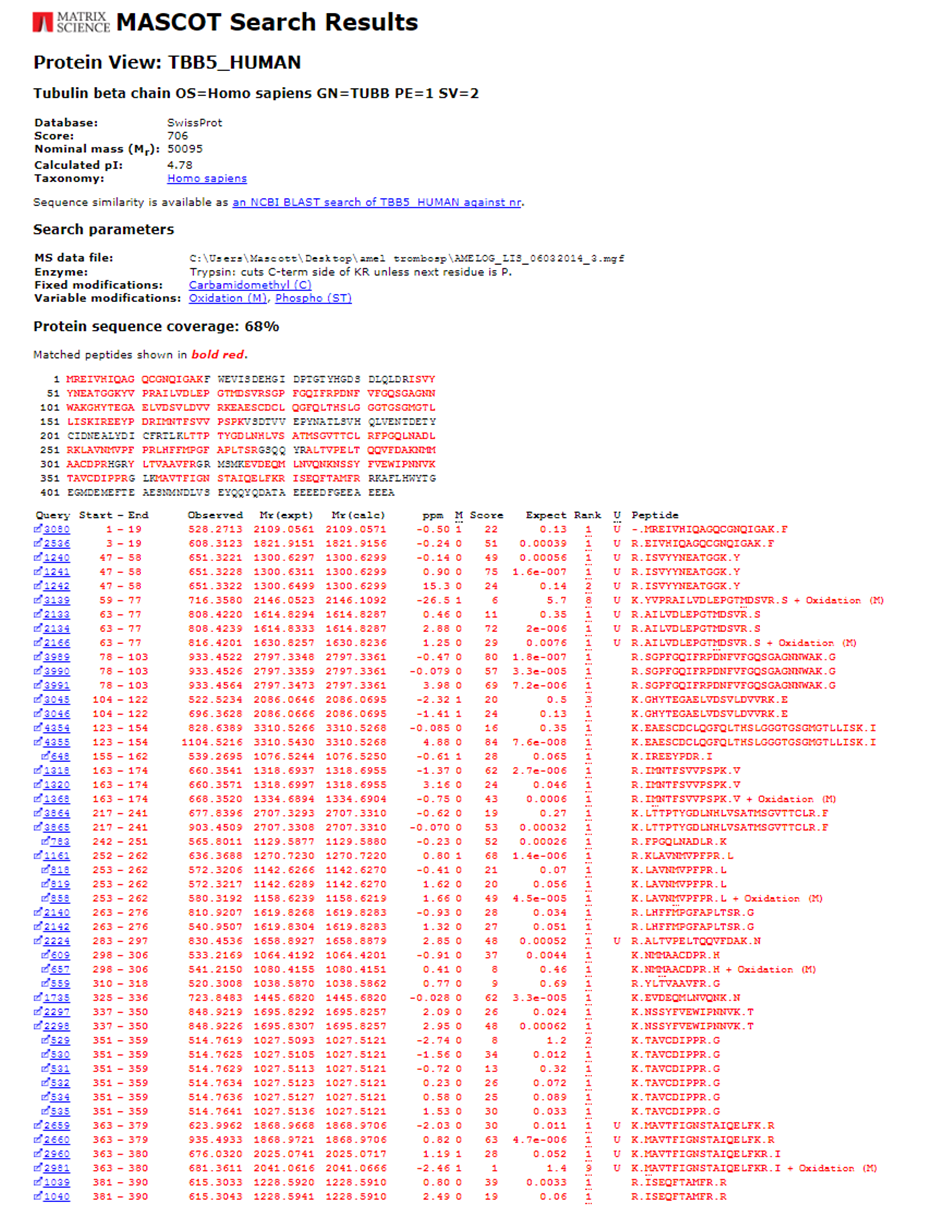
**

**
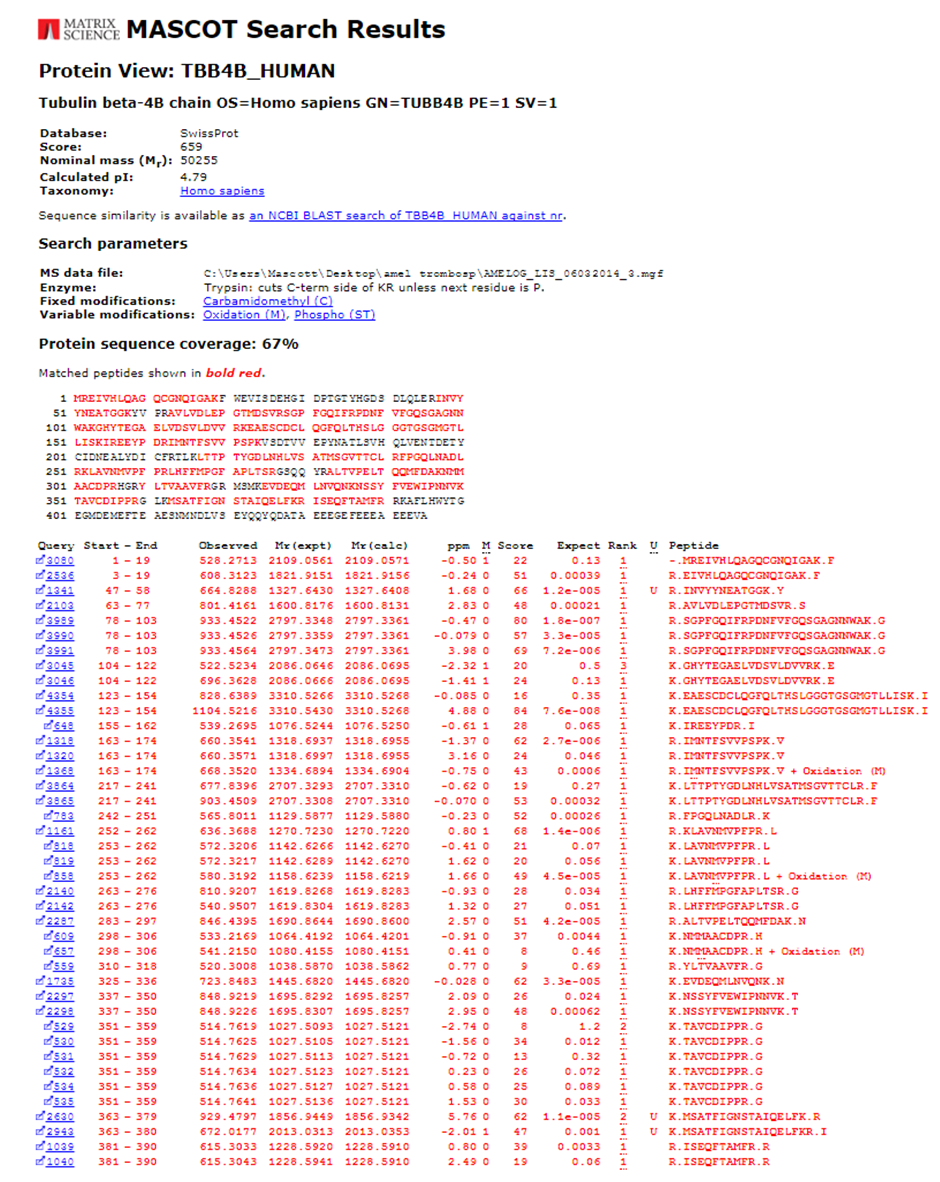
**

**
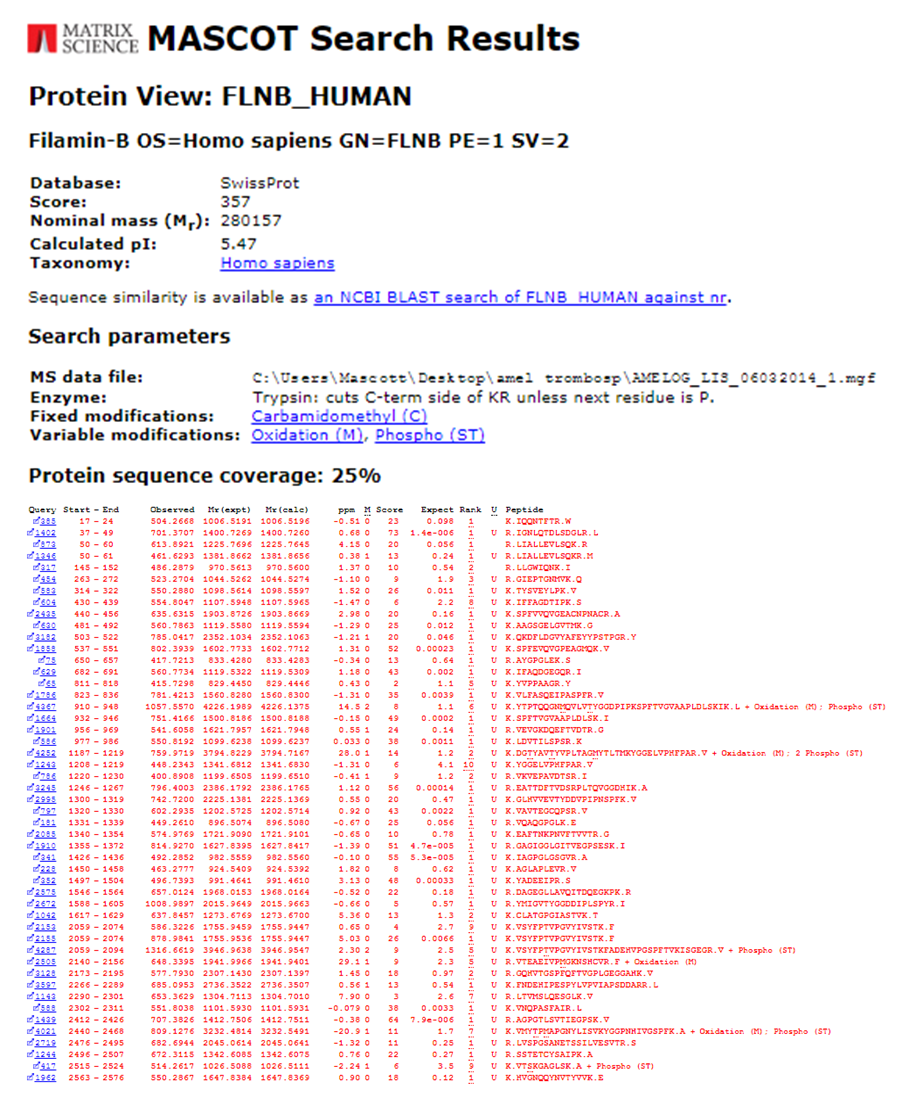
**

**
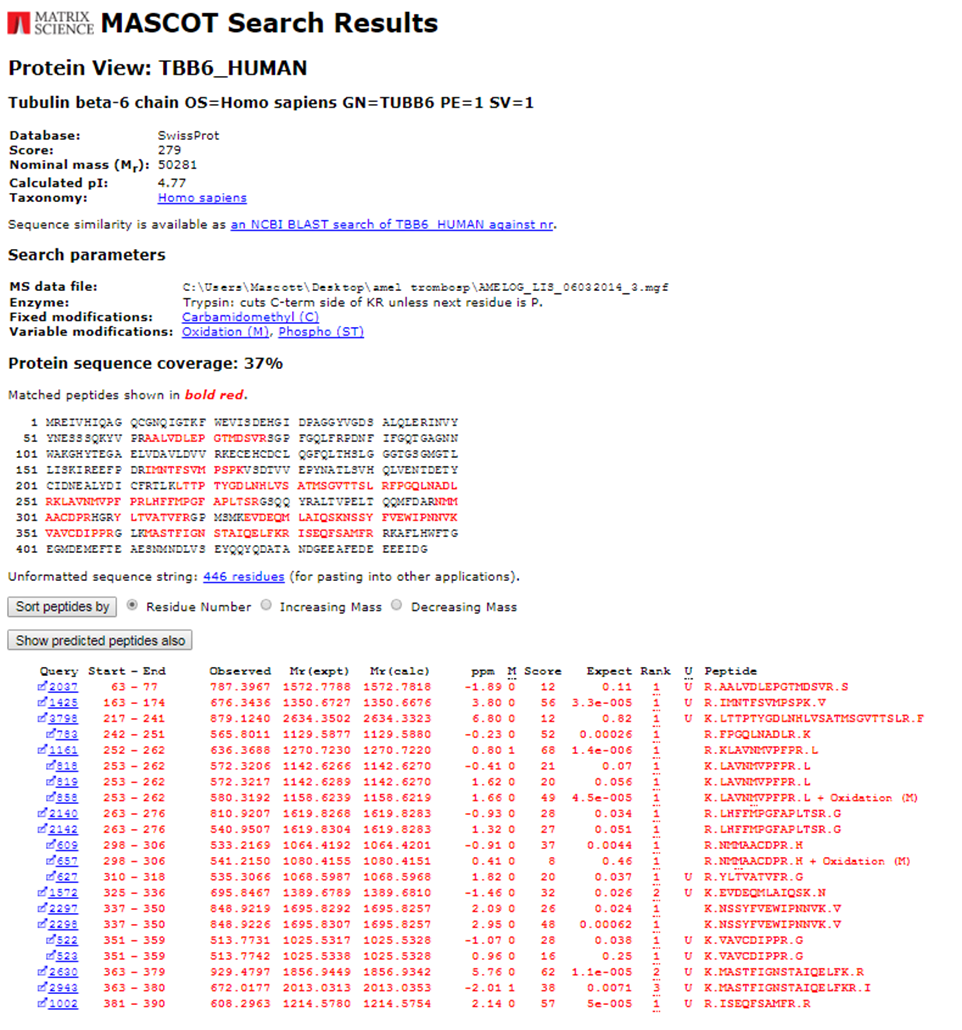
**

**
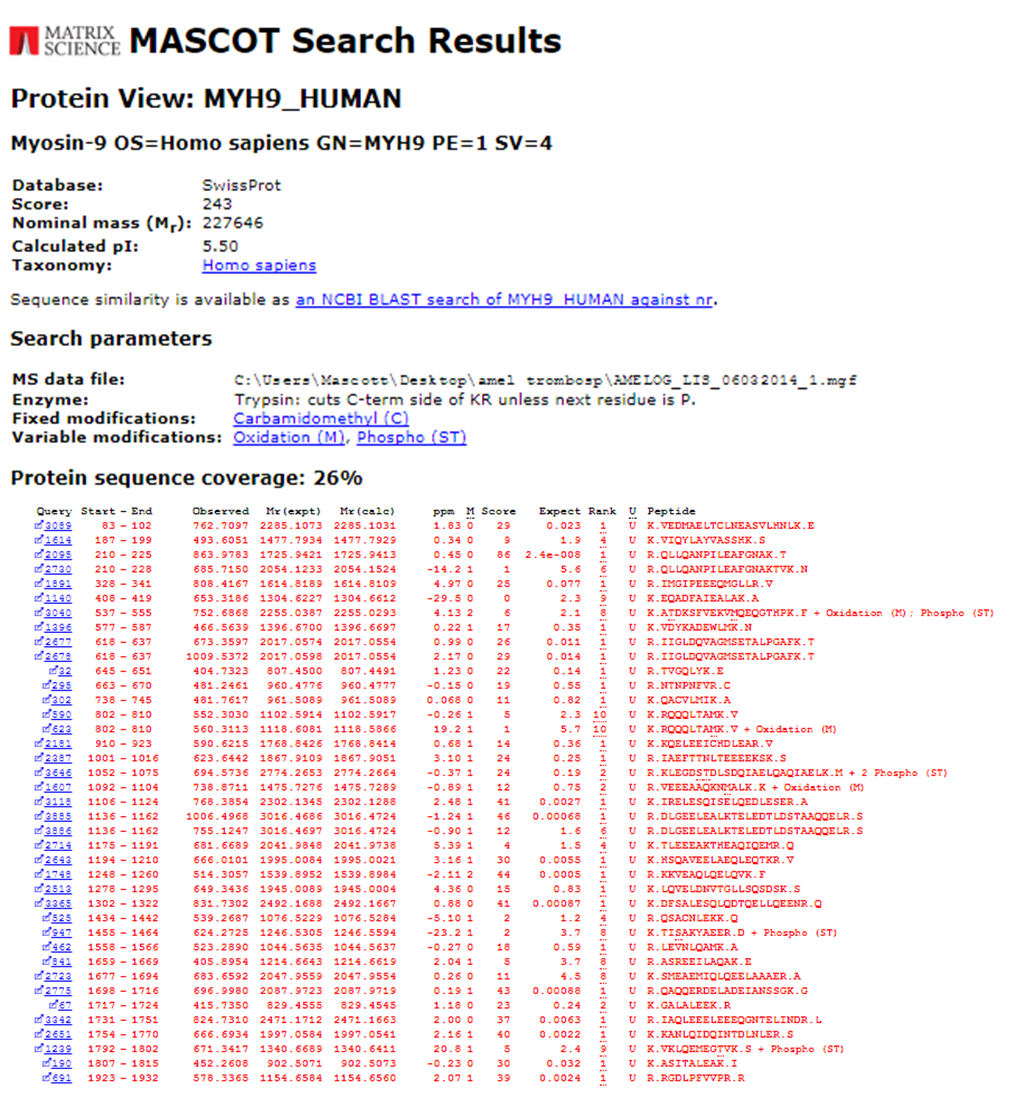

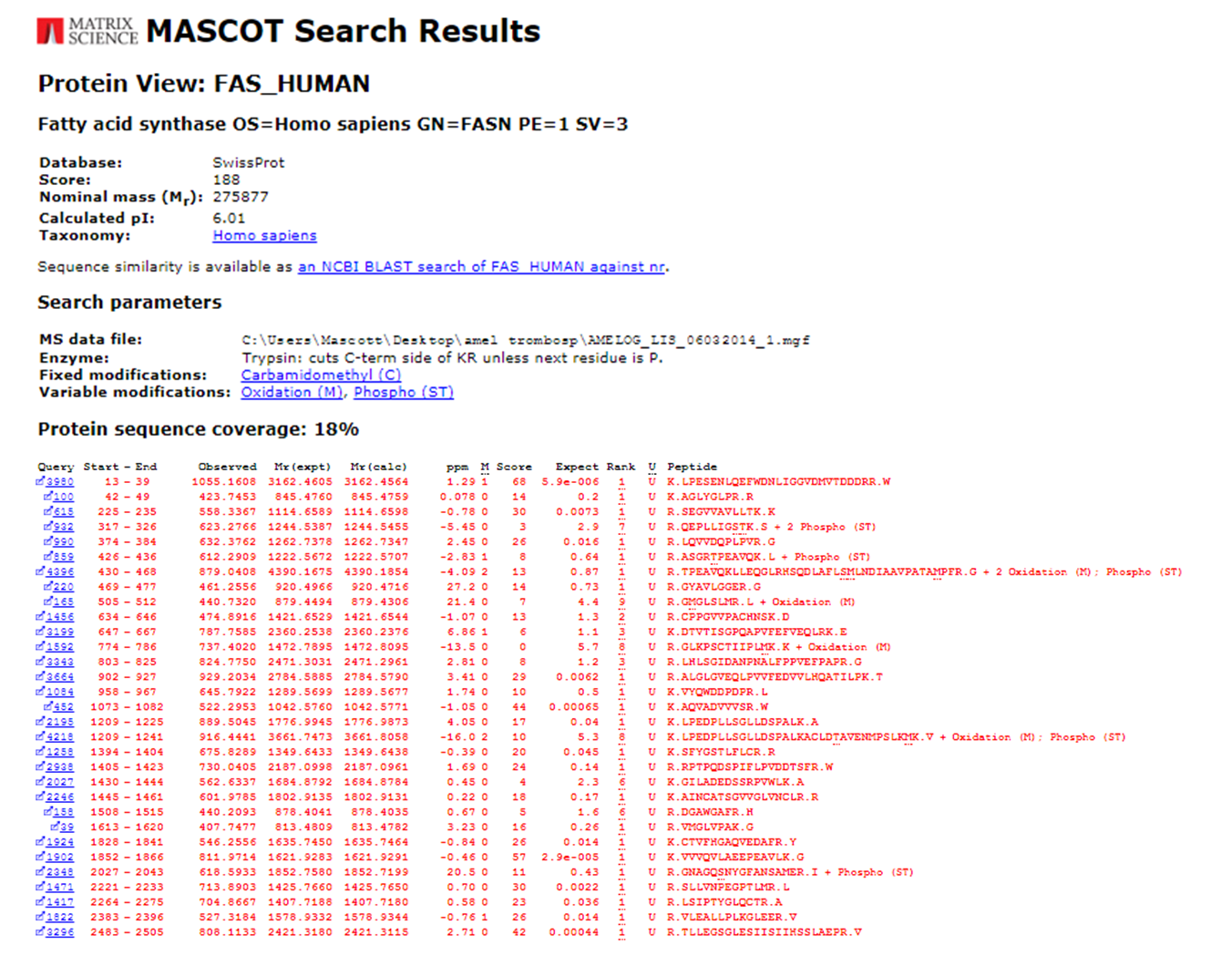

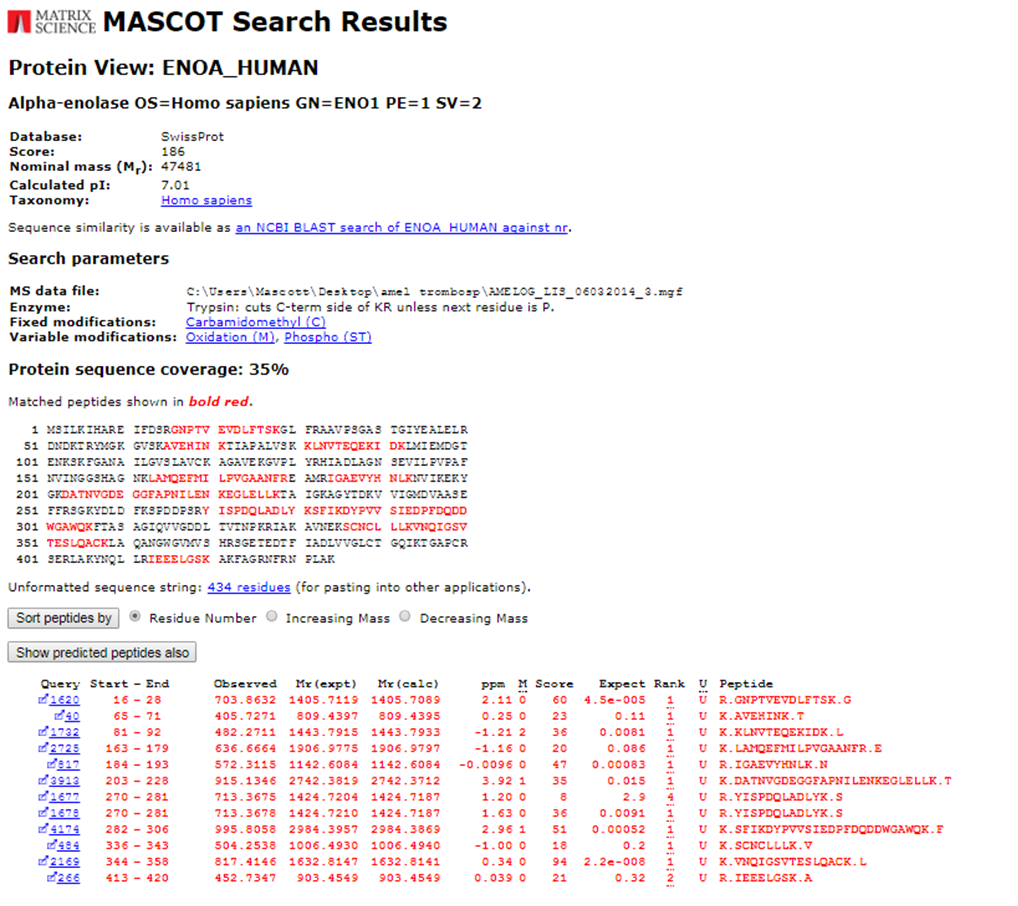
**

**
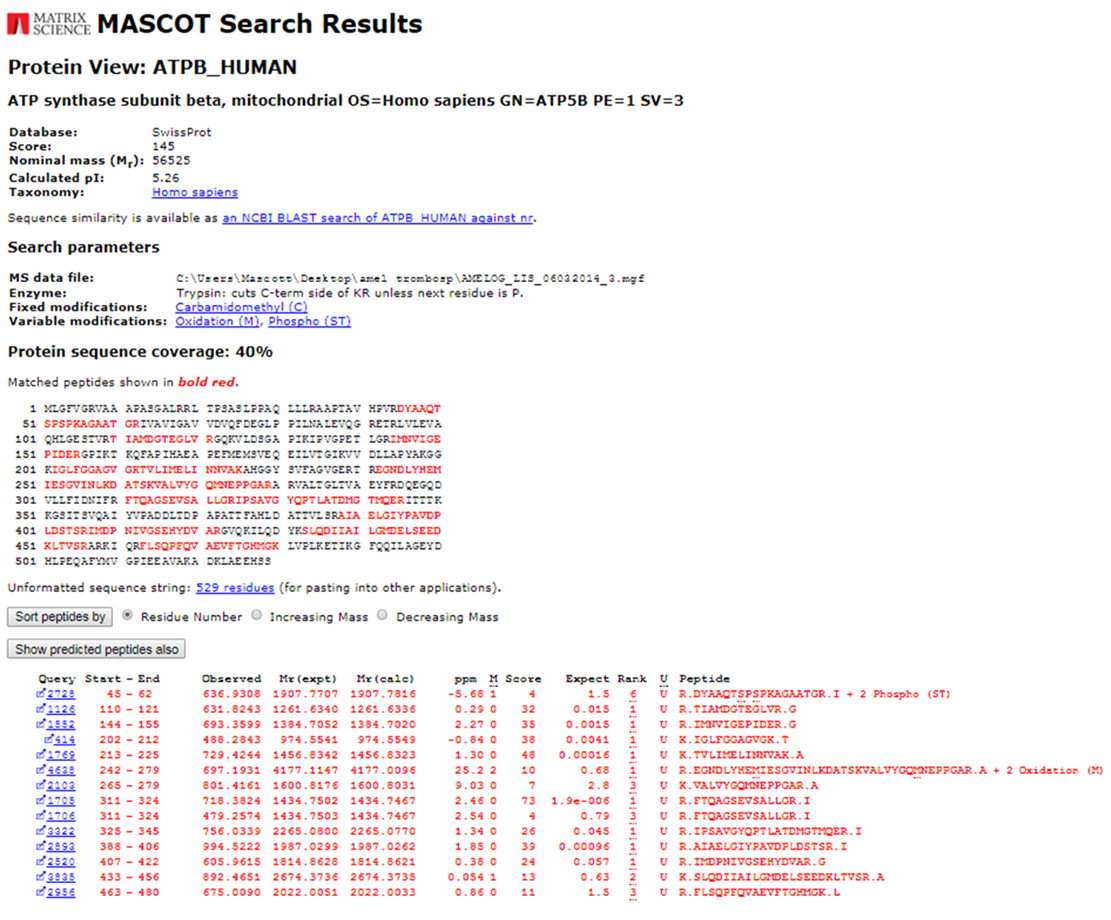

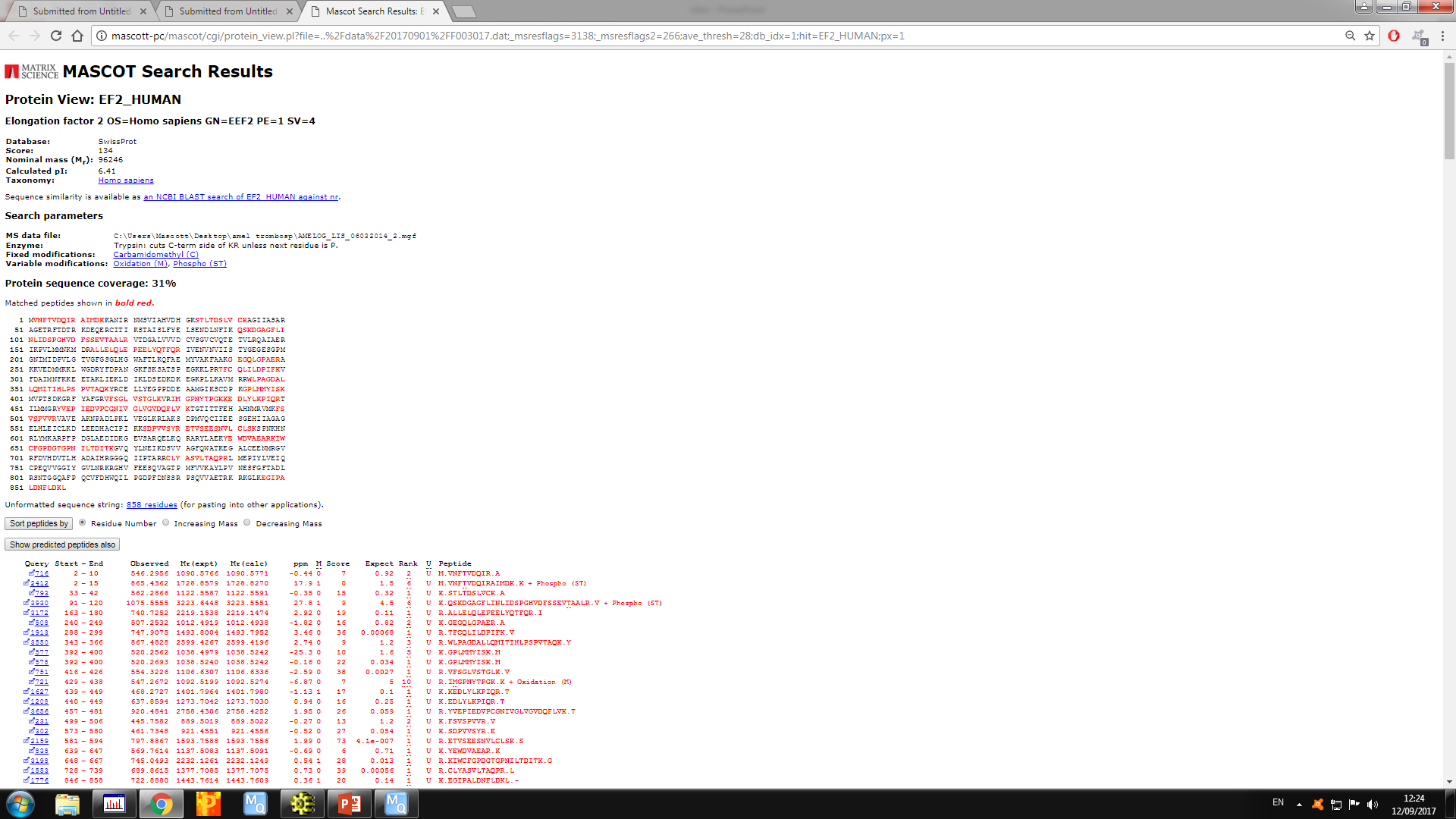
**

**
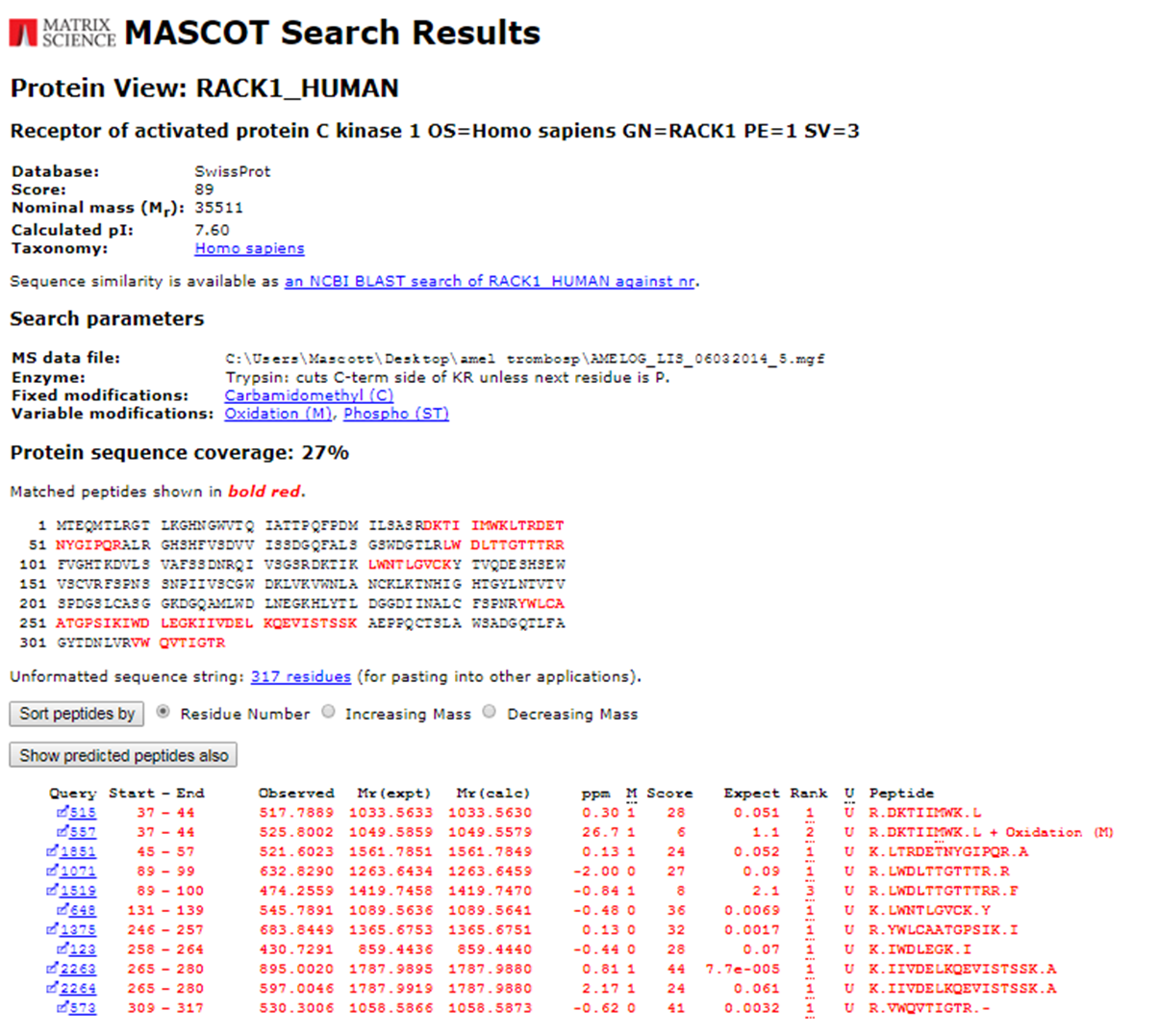
**

**
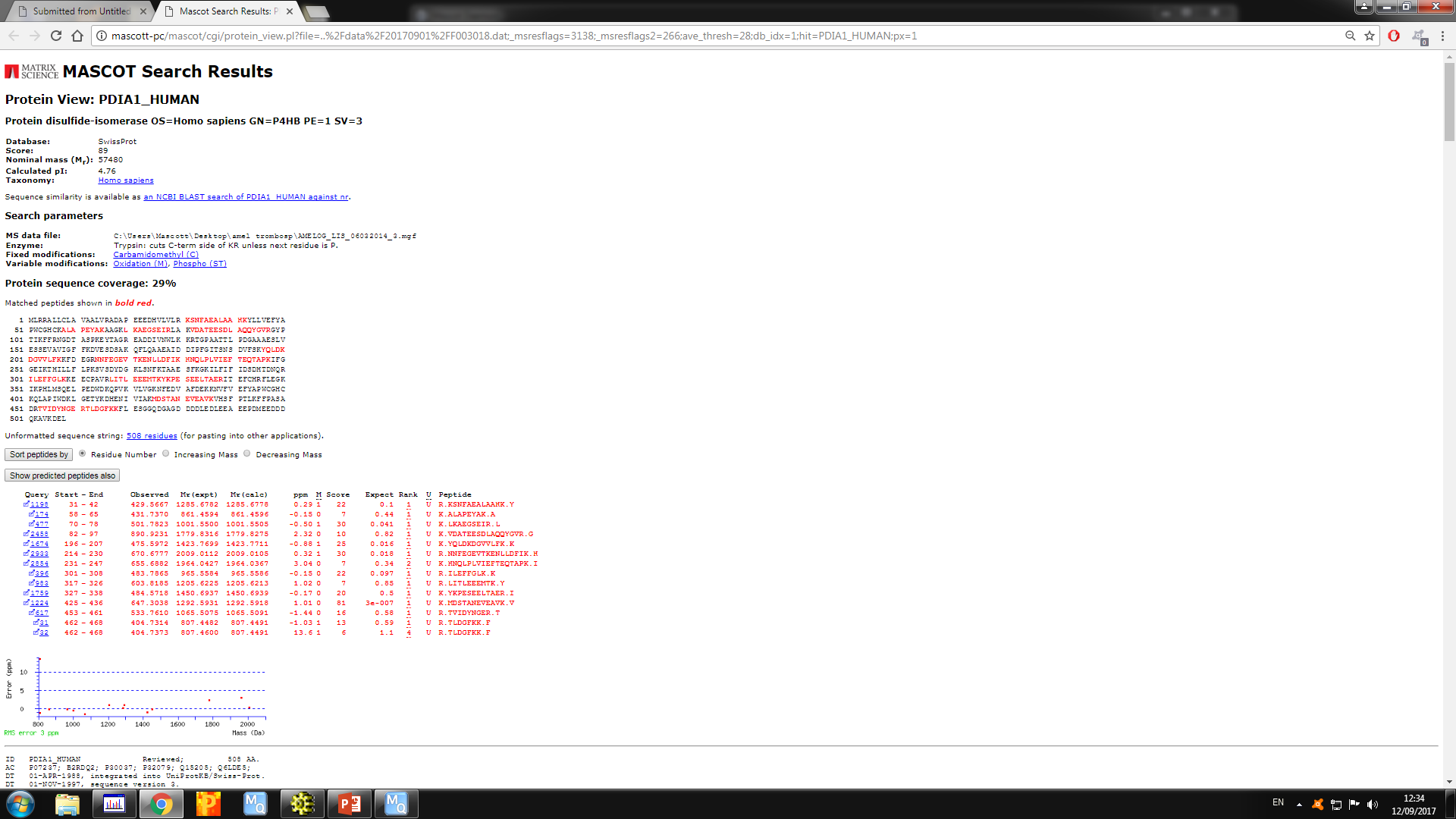
**

**
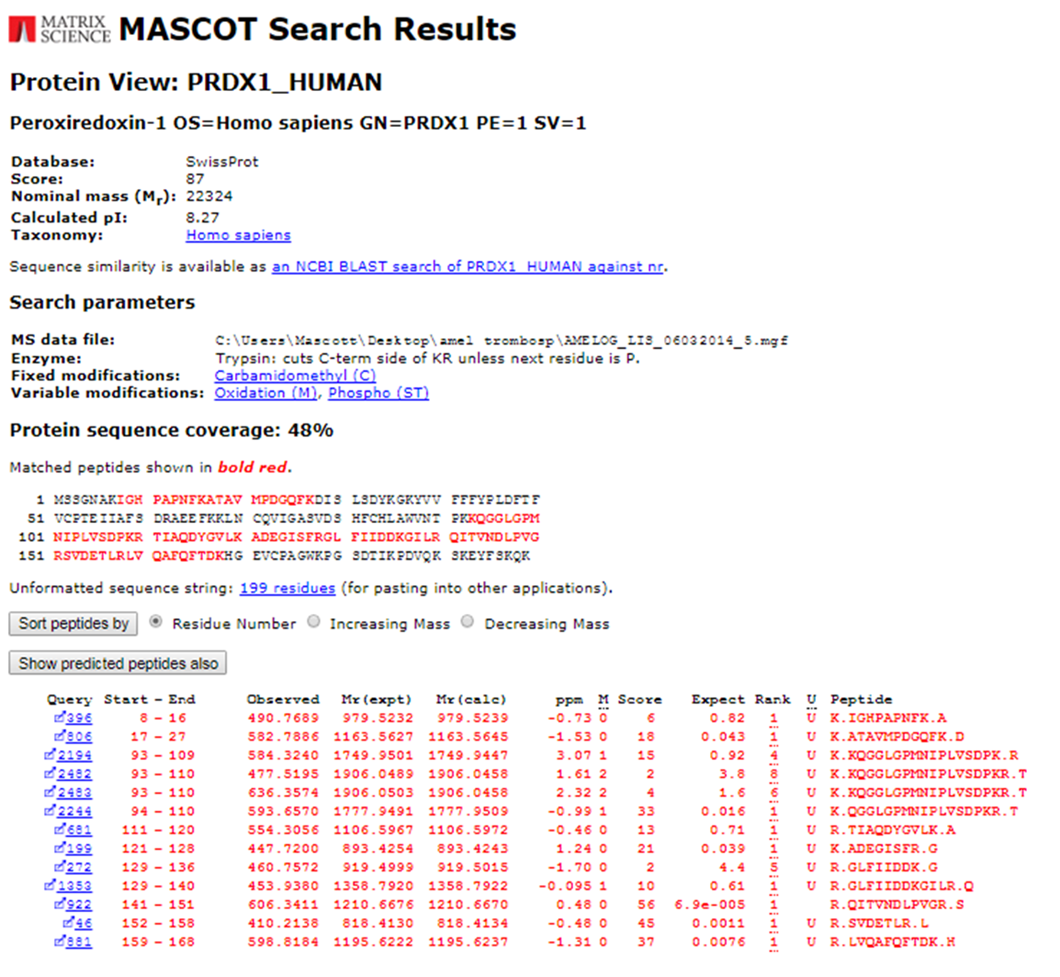
**

**
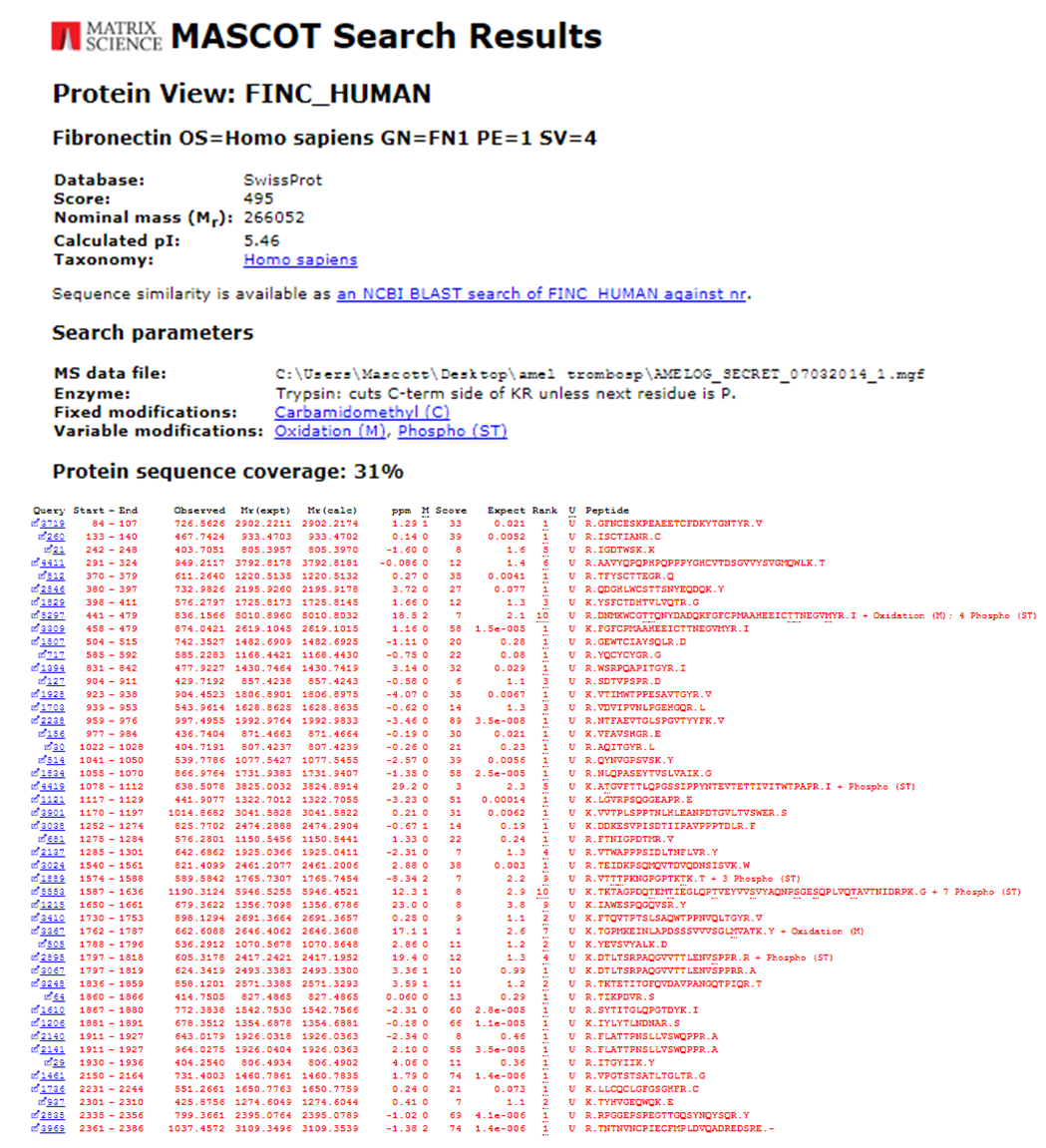
**

**Identification of TSP-1 HUMAN by Mascot Search together with all peptides fragmentation**

**
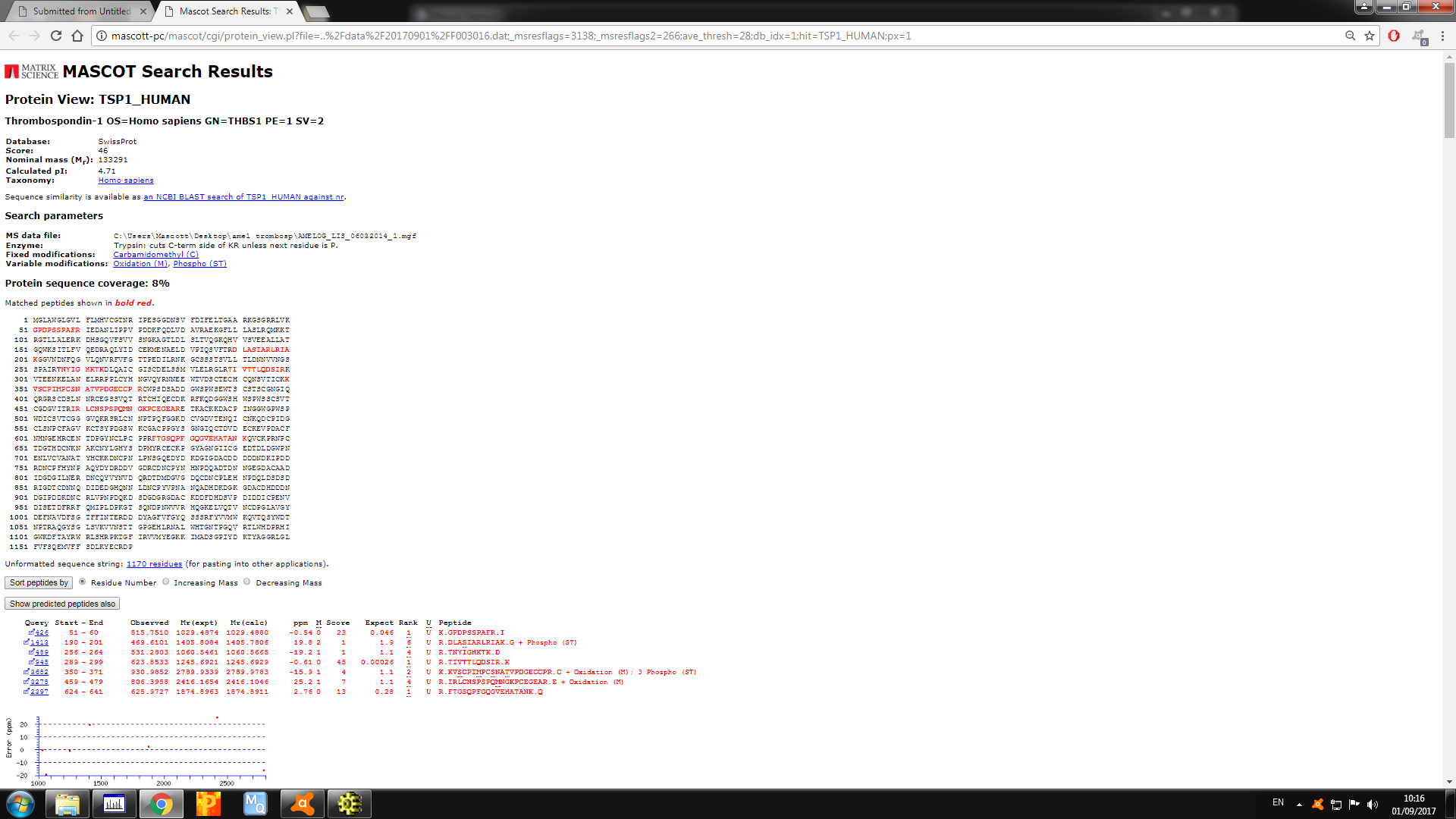
**


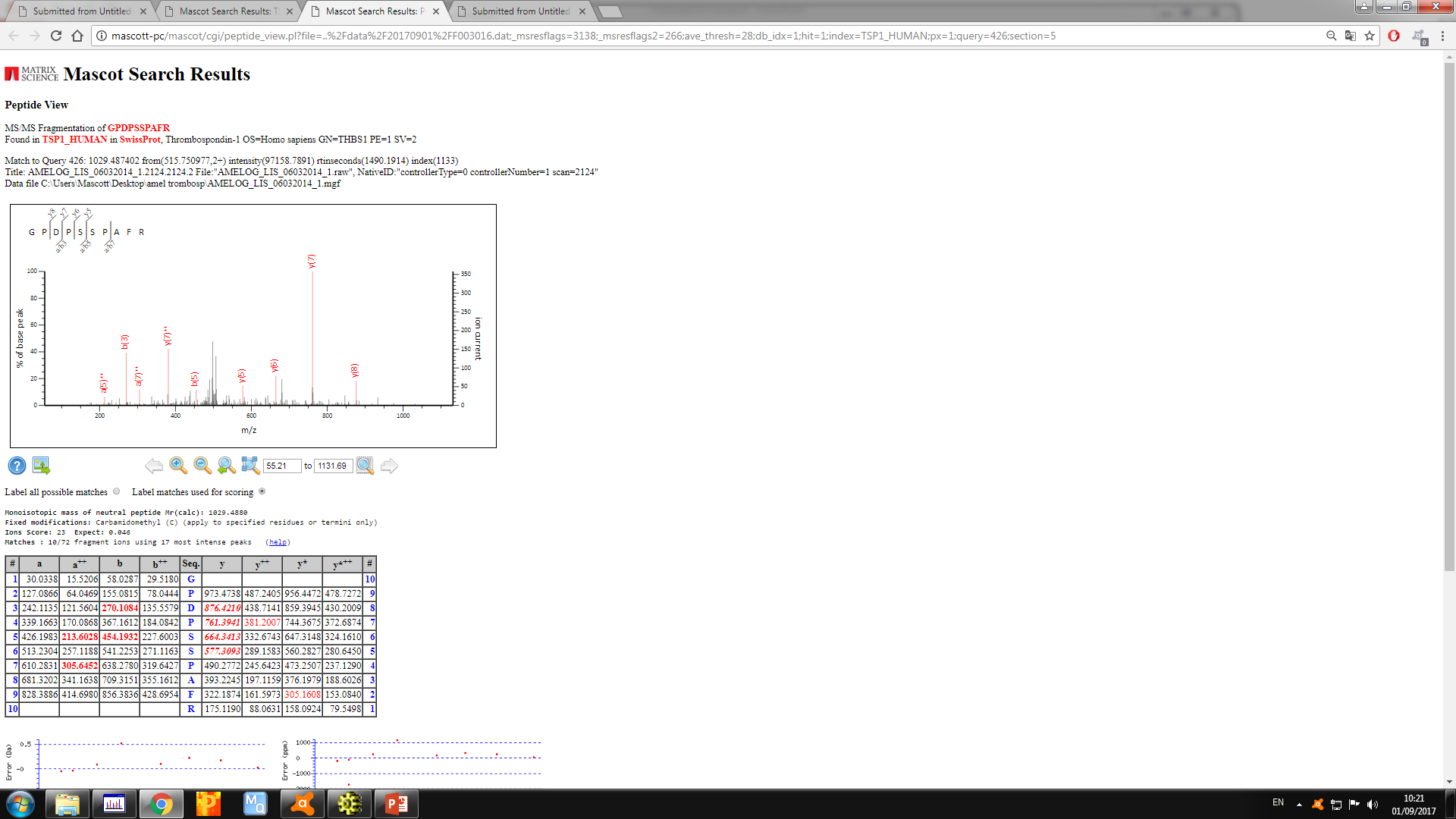


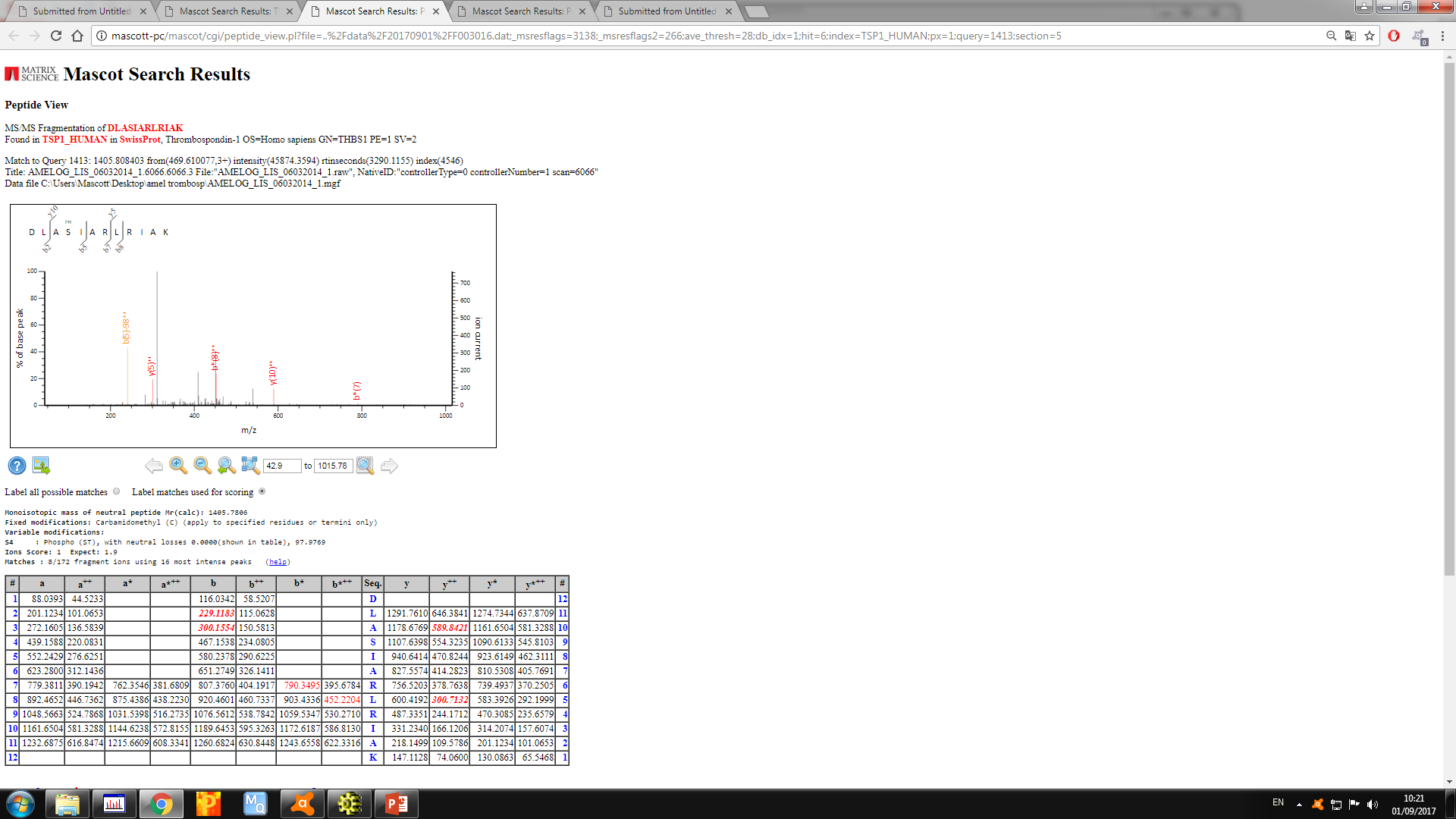


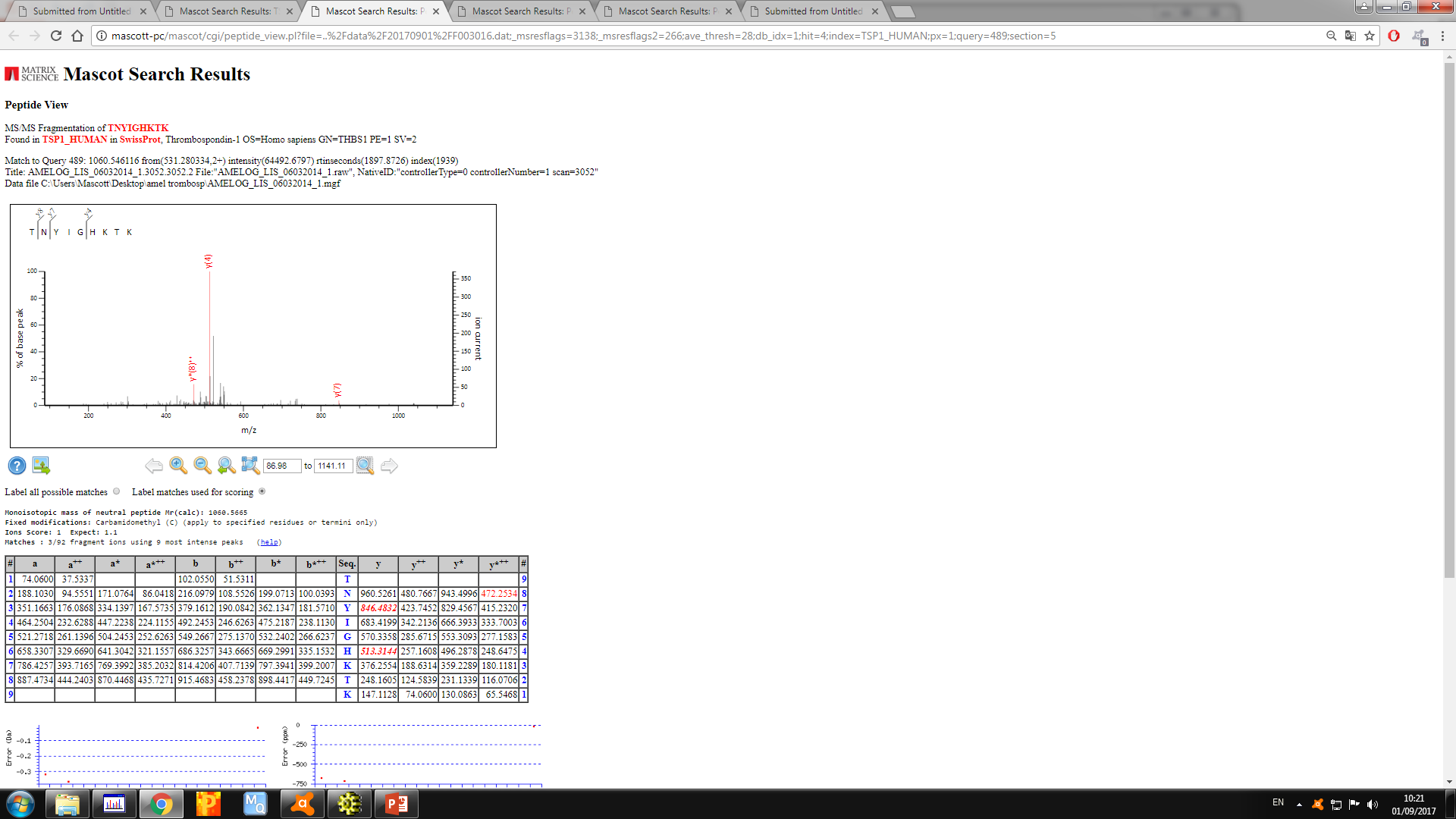


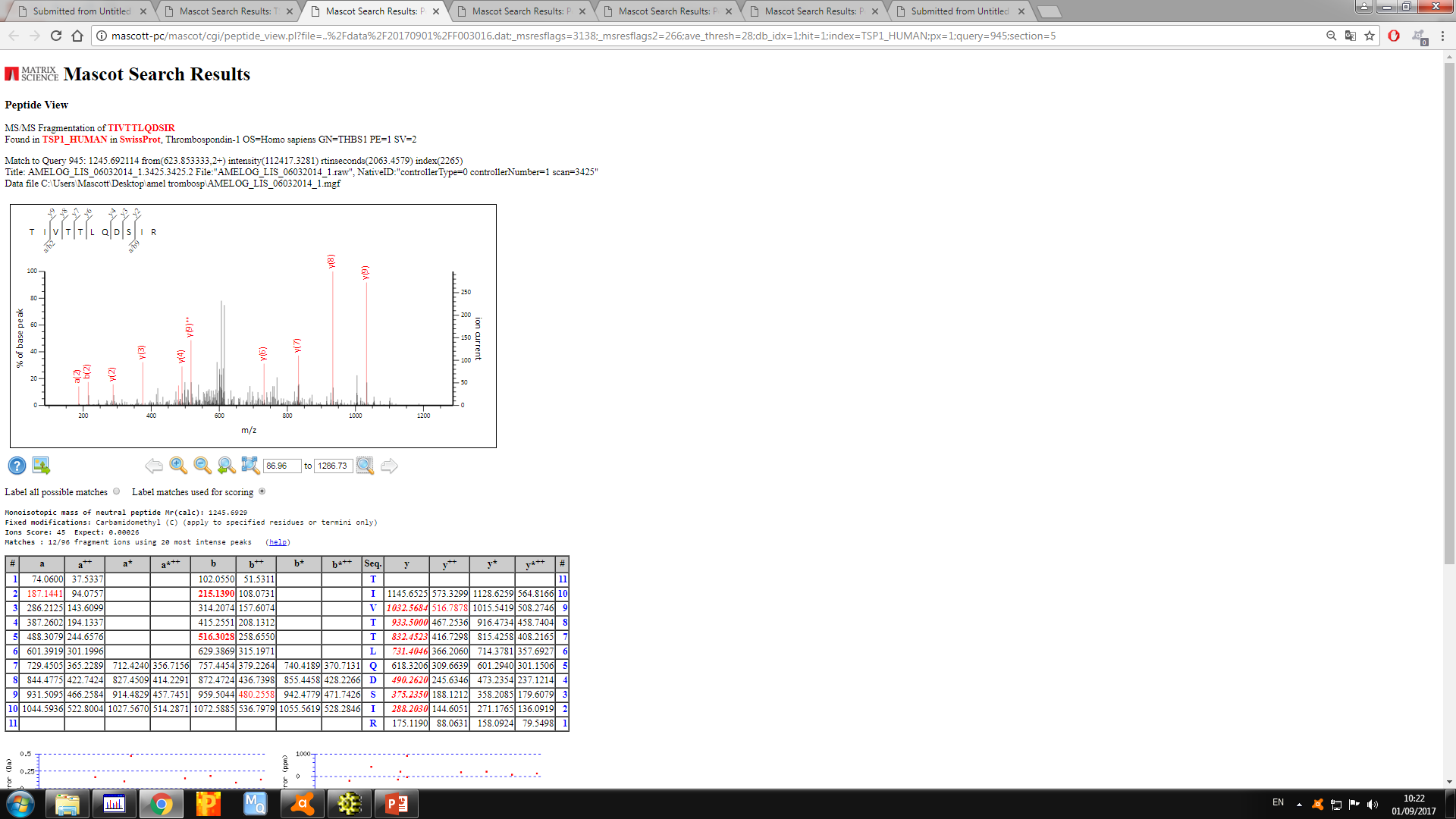


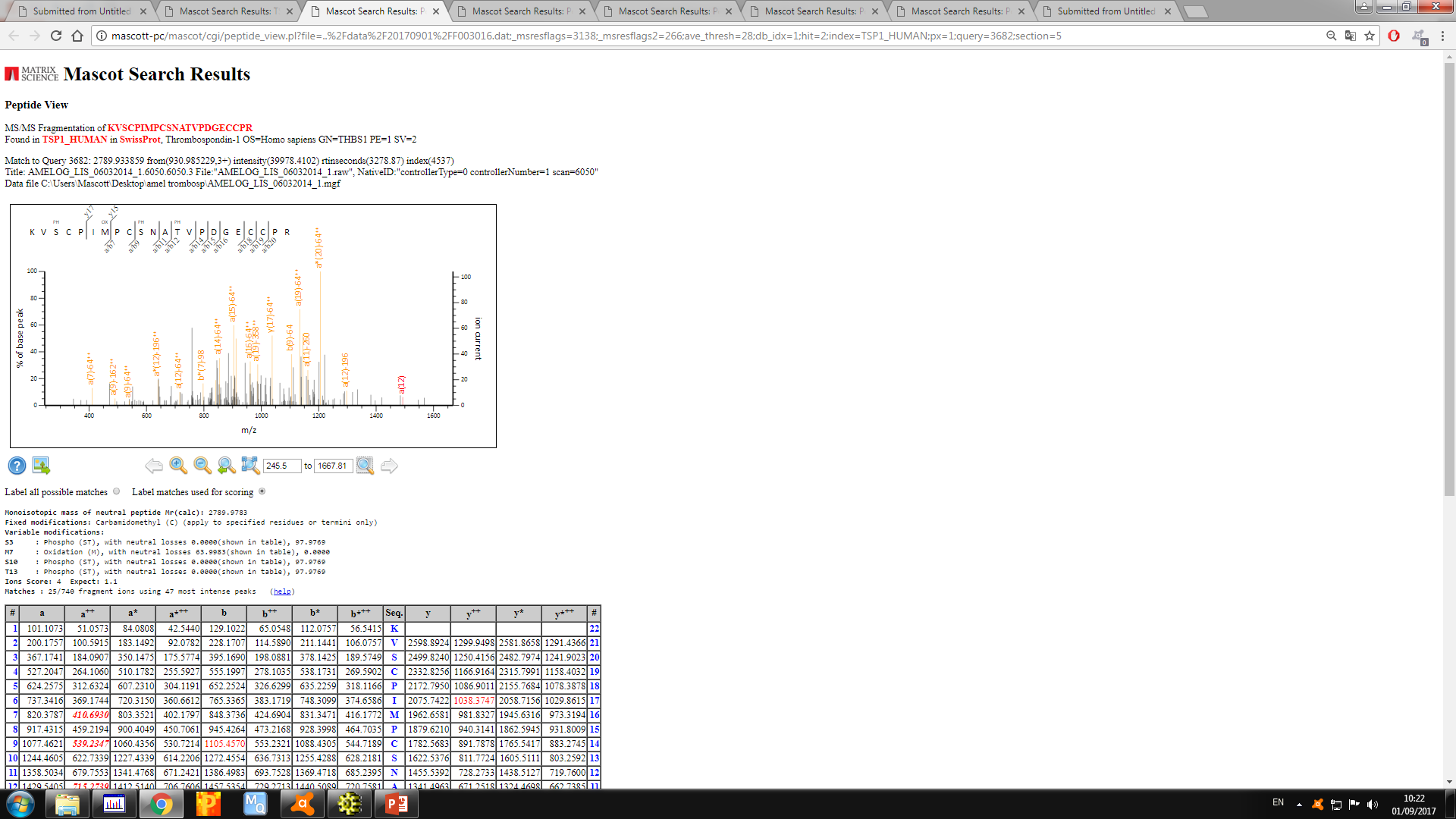


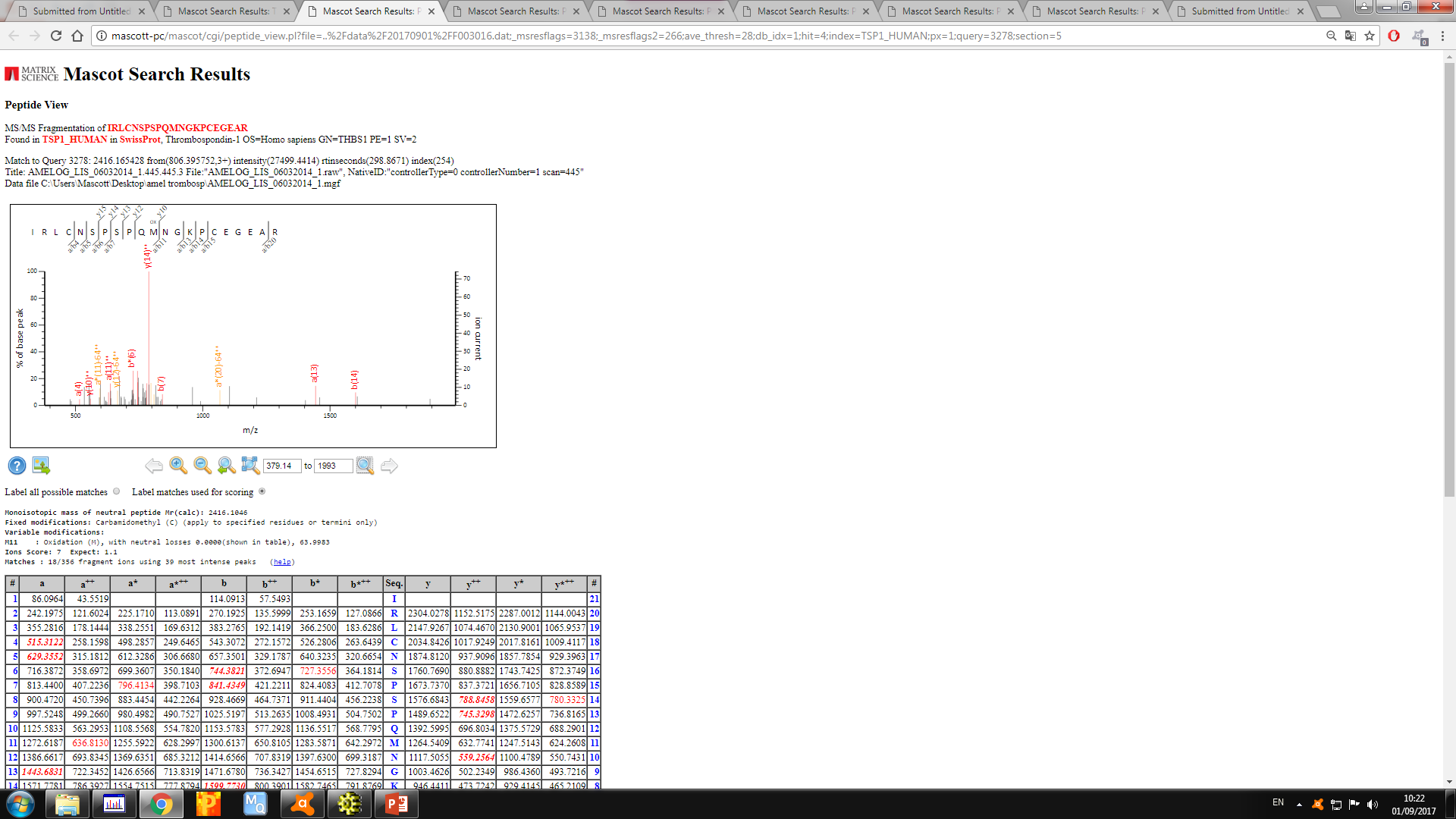


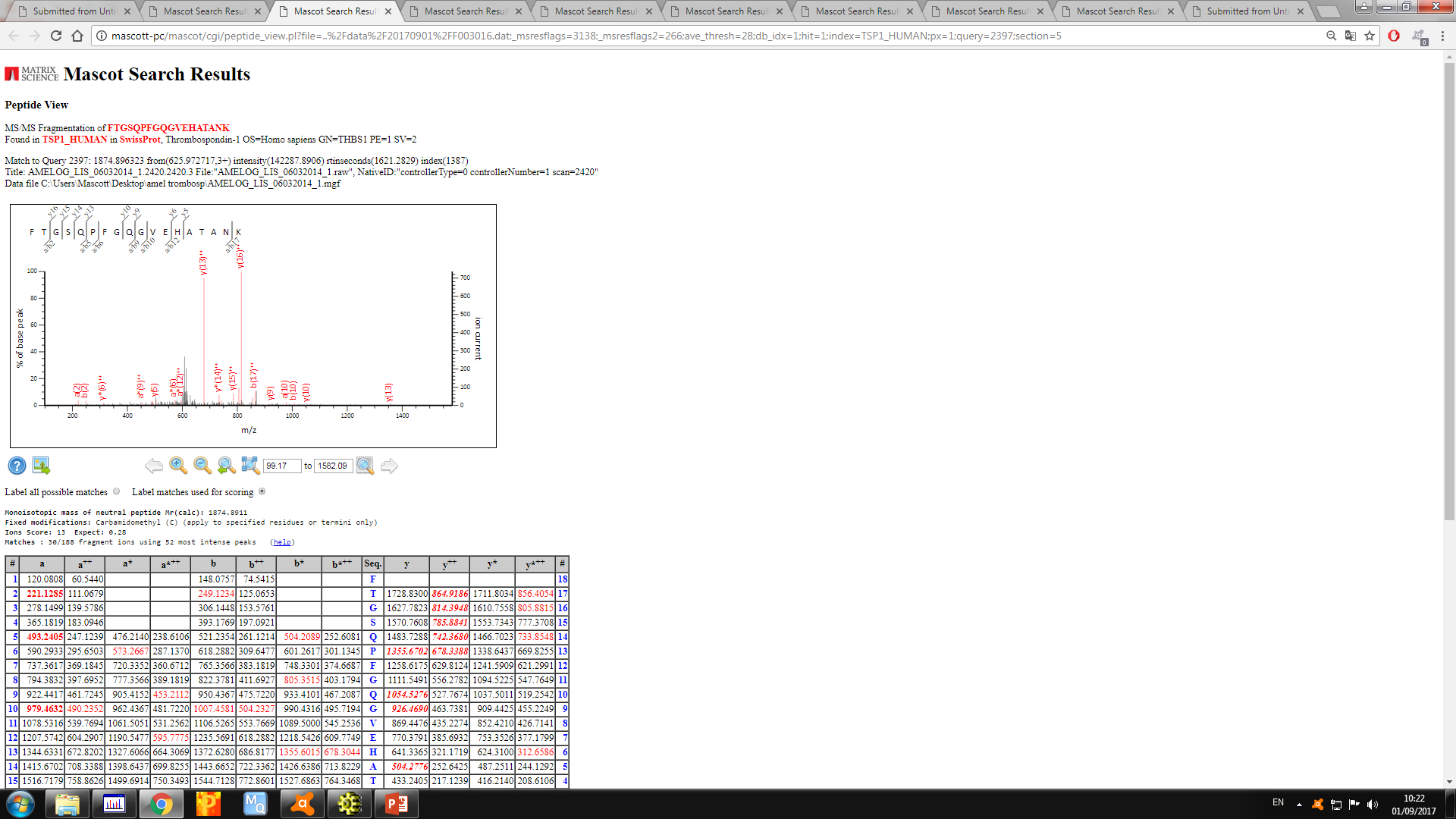

Supplement: Supplementary file 1 [file Table1.DOCX]
